# Dendritic Integration of Sensory Evidence in Perceptual Decision-Making

**DOI:** 10.1016/j.cell.2018.03.075

**Published:** 2018-05-03

**Authors:** Lukas N. Groschner, Laura Chan Wah Hak, Rafal Bogacz, Shamik DasGupta, Gero Miesenböck

**Affiliations:** 1 Centre for Neural Circuits and Behaviour, University of Oxford, Tinsley Building, Mansfield Road, Oxford OX1 3SR, UK; 2 MRC Brain Network Dynamics Unit, University of Oxford, Mansfield Road, Oxford OX1 3TH, UK

**Keywords:** membrane biophysics, synaptic integration, olfaction, decision-making, drift-diffusion model, neural integrator, potassium channel, forkhead box P transcription factors, *Drosophila melanogaster*

## Abstract

Perceptual decisions require the accumulation of sensory information to a response criterion. Most accounts of how the brain performs this process of temporal integration have focused on evolving patterns of spiking activity. We report that subthreshold changes in membrane voltage can represent accumulating evidence before a choice. αβ core Kenyon cells (αβ_c_ KCs) in the mushroom bodies of fruit flies integrate odor-evoked synaptic inputs to action potential threshold at timescales matching the speed of olfactory discrimination. The forkhead box P transcription factor (FoxP) sets neuronal integration and behavioral decision times by controlling the abundance of the voltage-gated potassium channel Shal (K_V_4) in αβ_c_ KC dendrites. αβ_c_ KCs thus tailor, through a particular constellation of biophysical properties, the generic process of synaptic integration to the demands of sequential sampling.

## Highlights

• Synaptic integration can implement a process of bounded evidence accumulation
• FoxP acts in αβ_c_ Kenyon cells (KCs) to repress the voltage-gated potassium channel Shal
• Shal regulates the ability of αβ_c_ KCs to summate synaptic inputs over time
• Altering Shal currents in αβ_c_ KCs changes spike latencies and decision times

## Graphical Abstract

**Figure:**
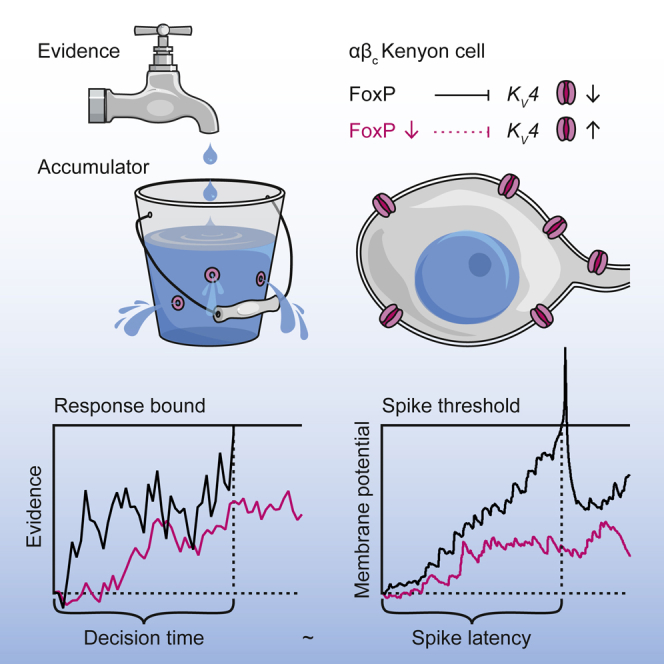

## Teaser

How is information integrated to allow perceptual decision-making? FoxP-expressing neurons in fruit flies accumulate sensory evidence as subthreshold voltage changes. The first spike signals that a decision.

## Introduction

Decisions take time because the information needed to make them is rarely available all at once but must be gathered sequentially. A large literature, whose beginnings stretch back to the 19^th^ century (Donders, 1869), documents systematic variations in the speed of perceptual judgments with stimulus strength: easy decisions, based on unambiguous evidence, tend to be fast, whereas difficult decisions, based on weak or conflicting data, tend to be slow (Hanes and Schall, 1996, Roitman and Shadlen, 2002, Shadlen and Newsome, 2001, Vickers, 1970). This difficulty-dependent cost of decision time likely reflects the need to construct time-averaged sensory representations. Just as engineers average signals over time to reduce the effects of contaminating noise, the brain appears to improve its signal-to-noise ratio by integrating information from sequential samples. The neural structures and mechanisms responsible for the execution and termination of this integration process remain incompletely understood (Gold and Shadlen, 2007, Shadlen and Kiani, 2013).

From first principles, accumulating information must be encoded in time-varying neural signals that span the evidence-gathering period. Smooth ramps or discrete steps in firing frequency or domino-like activity sequences, which can all be observed before choices are made, have been interpreted to represent these signals (Gold and Shadlen, 2007, Hanes and Schall, 1996, Harvey et al., 2012, Latimer et al., 2015, Roitman and Shadlen, 2002, Shadlen and Newsome, 2001, Shadlen and Kiani, 2013). The decision is considered complete when the mean spike rate in the population crosses a threshold or the activity sequence joins a choice-specific trajectory. However, there is little evidence that changing the slope of a ramp or the order or speed at which activity propagates through a sequence alters decision times. And although mechanisms for producing the requisite spiking dynamics have been advanced theoretically (Rajan et al., 2016, Wang, 2002), experimental support for these mechanisms has been difficult to obtain.

Like mammals, fruit flies take longer to commit to difficult perceptual choices than to easy ones (DasGupta et al., 2014), and the same quantitative relationships—formalized by drift-diffusion models of evidence accumulation (Bogacz et al., 2006, Ratcliff, 1978, Ratcliff and McKoon, 2008)—link speed, accuracy, and task difficulty. When flies discriminate between two odor concentrations, the amount of time taken is influenced by a small, genetically distinct (Aso et al., 2014a, Tanaka et al., 2008) population of third-order olfactory neurons: the αβ core Kenyon cells (αβ_c_ KCs) of the mushroom bodies (DasGupta et al., 2014). The decision-relevant αβ_c_ KCs encompass just ∼160 of the 100,000 neurons in the fly brain and are distinguished by the expression of the forkhead box P transcription factor (FoxP) (DasGupta et al., 2014), an ancestral member (Santos et al., 2011) of a transcription factor family of considerable interest in molecular and developmental neurobiology, cognitive science, linguistics, and evolutionary anthropology (Enard et al., 2002, Hamdan et al., 2010, Konopka et al., 2009, Lai et al., 2001, Vargha-Khadem et al., 1995). *Drosophila FoxP* mutants are slower to make difficult odor intensity discriminations than wild-type flies, and, in some allelic combinations, are also more error prone (DasGupta et al., 2014). Here, we study the mechanism of sequential sampling through the prism of the *FoxP* mutation: we identify how FoxP shapes the integrative capabilities of αβ_c_ KCs and relate these capabilities to the decision-making behavior of the animal.

## Results

### Biophysics of FoxP-Positive and FoxP-Negative Kenyon Cells

**Figure 1:**
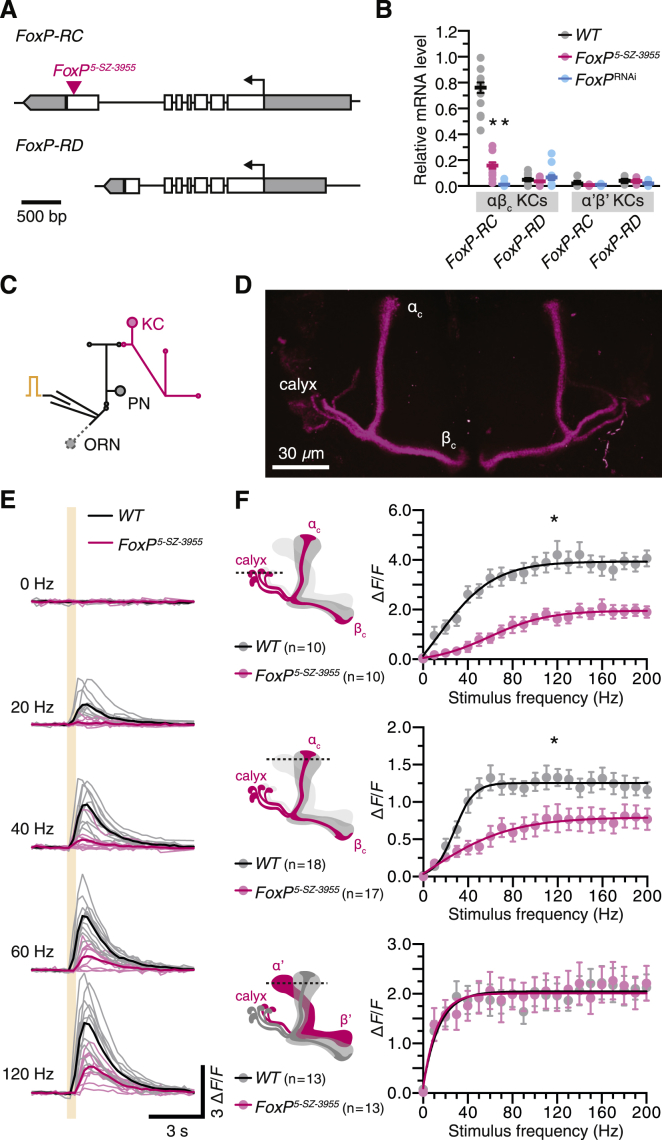
FoxP Controls the Responsiveness of αβ_c_ KCs to Antennal Nerve Stimulation (A) The P element insertion (red) in the mutant *FoxP*^*5-SZ-3955*^ allele maps to the alternatively spliced exon of the *FoxP-RC* isoform. (B) Levels of polysome-bound *FoxP* mRNA in αβ_c_ or α’β’ KCs of wild-type (WT) flies (black), homozygous *FoxP*^*5-SZ-3955*^ mutants (red), or flies expressing *FoxP*^RNAi^ (blue), relative to the geometric mean of three marker gene transcripts (circles, biological replicates; bars, means ± SEM; see Table S1 for sample sizes). One-way ANOVA detected a significant genotype effect on *FoxP-RC* levels in αβ_c_ KCs (p < 0.0001); asterisks denote significant differences from wild-type in post hoc comparisons. (C) Antennal nerve stimulation and two-photon imaging *in vivo*. ORN, olfactory receptor neuron; PN, projection neuron. (D) *NP7175-GAL4*-driven GCaMP6m expression in αβ_c_ KCs. (E) Ca^2+^ transients in αβ_c_ KC dendrites evoked by 0.5 s epochs of antennal nerve stimulation at the indicated frequencies of individual wild-type (black, n = 10) or homozygous *FoxP*^*5-SZ-3955*^ mutant flies (red, n = 10). Solid traces represent group averages. (F) Peak *ΔF/F* as a function of stimulation frequency in αβ_c_ KC dendrites (calyx, top) or axons (center) or in α’β’ KC axons (bottom) of wild-type (black) or homozygous *FoxP*^*5-SZ-3955*^ mutant flies (red). Dashed lines in the schematics mark the approximate positions of the imaging planes. Data are means ± SEM. Asterisks indicate significant differences between the stimulus-response curves of wild-type and *FoxP*^*5-SZ-3955*^ mutant flies (p < 0.0001, *F* test). See also Figures S1 and S2 and Tables S1 and S5.

**Figure S1:**
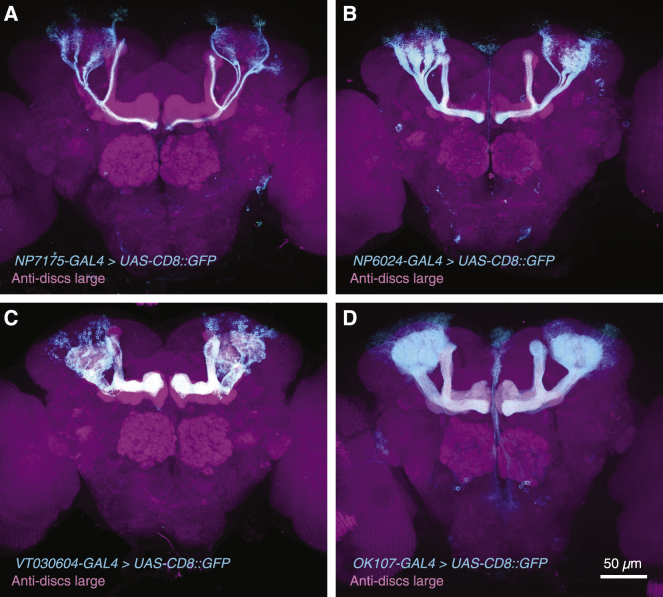
Expression Patterns of GAL4 Lines, Related to Figure 1 (A–D) Maximum intensity projections of confocal image stacks of the central brains of flies expressing *UAS-CD8::GFP* (cyan) under the control of *NP7175-GAL4* (A) or *NP6024-GAL4* (B) in αβ_c_ KCs; under the control of *VT030604-GAL4* in α’β’ KCs (C); or under the control of *OK107-GAL4* in all KCs (D). Synaptic structures were counterstained with an antibody against discs large (magenta).

The single *FoxP* gene of *Drosophila* consists of six constitutively and two alternatively spliced exons that give rise to a minimum of two transcripts (*RC* and *RD*) and corresponding protein isoforms (Santos et al., 2011) (Figure 1A). The two isoforms share an N-terminal zinc finger domain but possess different C-terminal forkhead domains. The transposable element insertion sites of mutant *FoxP* alleles associated with reaction time phenotypes (DasGupta et al., 2014), including *FoxP*^*5-SZ-3955*^, map to the last, alternatively spliced exon and are therefore predicted to disrupt the *RC* transcript (Figure 1A). To test this prediction, we used *NP7175-GAL4* or *NP6024-GAL4* to drive moderate to high levels of a GFP-tagged ribosomal protein (Heiman et al., 2008, Huang et al., 2013) in αβ_c_ KCs (Aso et al., 2009, Murthy et al., 2008, Tanaka et al., 2004, Tanaka et al., 2008) (Figures S1A and S1B). These neurons are also marked, albeit more weakly, by a *FoxP* promoter fragment (DasGupta et al., 2014). Immunoprecipitation of tagged ribosomes from head homogenate (translating ribosome affinity purification or TRAP) (Heiman et al., 2008), followed by reverse transcription of polysome-bound mRNA and quantitative real-time PCR (qPCR), revealed an abundance of *FoxP-RC* (but not *FoxP-RD*) transcript in αβ_c_ neurons (Figure 1B). *FoxP-RC* mRNA levels were greatly reduced in homozygous *FoxP*^*5-SZ-3955*^ mutants and flies expressing *NP7175-GAL4*-driven hairpin RNA directed against the *FoxP* transcript (*FoxP*^RNAi^) (Figure 1B). α’β’ neurons, which lie outside the *FoxP-GAL4* expression domain (DasGupta et al., 2014) but could be captured by the *VT030604-GAL4* driver (Figure S1C), contained only trace amounts of both *FoxP* isoforms, regardless of genotype (Figure 1B). The mushroom body thus represents a mosaic of FoxP-positive and FoxP-negative KCs, as suggested by the expression pattern of *FoxP-GAL4* (DasGupta et al., 2014).

**Figure S2:**
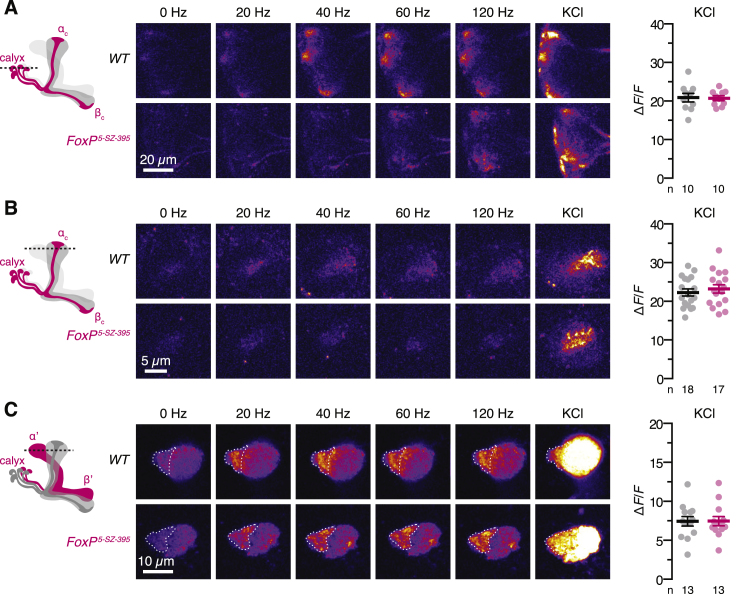
GCaMP6m Signals in KCs, Related to Figure 1 (A–C) Raw two-photon images of GCaMP6m fluorescence in dendrites (A) or axons (B) of αβ_c_ KCs or axons of α’β’ KCs (circled regions of interest in C) at the indicated stimulation frequencies, in wild-type flies (top) and homozygous *FoxP*^*5-SZ-3955*^ mutants (bottom). At the end of each imaging experiment, neurons were bulk-depolarized by elevating the extracellular KCl concentration to 100 mM (right). t tests failed to detect significant differences between the peak changes in GCaMP6m fluorescence evoked by 100 mM KCl in wild-type flies (black) and homozygous *FoxP*^*5-SZ-3955*^ mutants (red) (p ≥ 0.5245).

For an initial determination of whether and how FoxP regulates the function of neurons that express this transcription factor, we targeted the Ca^2+^ indicator GCaMP6m to FoxP-positive αβ_c_ or FoxP-negative α’β’ KCs and imaged Ca^2+^ transients evoked by electrical stimulation of the ipsilateral antennal nerve (Figures 1C–1E and S2). Electrical instead of odor stimulation allowed us to recruit many glomerular channels simultaneously and activate KCs broadly rather than in sparse odor-specific ensembles whose membership varies unpredictably from fly to fly (Caron et al., 2013, Gruntman and Turner, 2013, Murthy et al., 2008). The dendritic and axonal compartments of both types of KC responded in a saturating fashion to stimulus trains of increasing frequency, but the response amplitudes of FoxP-negative α’β’ cells increased more steeply and leveled off at lower stimulation intensities than those of FoxP-positive αβ_c_ KCs (Figures 1E and 1F). Mutating *FoxP* flattened the stimulus-response curves of αβ_c_ KCs and caused them to saturate at reduced plateaux (Figures 1E and 1F). Because the responses of α’β’ neurons were unaffected in mutants (Figure 1F), the overall organization and strength of inputs from olfactory projection neurons, which are thought to be shared among all classes of KC (Caron et al., 2013, Gruntman and Turner, 2013), appear preserved. Changes specific to the FoxP-positive αβ_c_ KCs must therefore lie at the root of the selective response attenuation of these cells.

**Figure 2:**
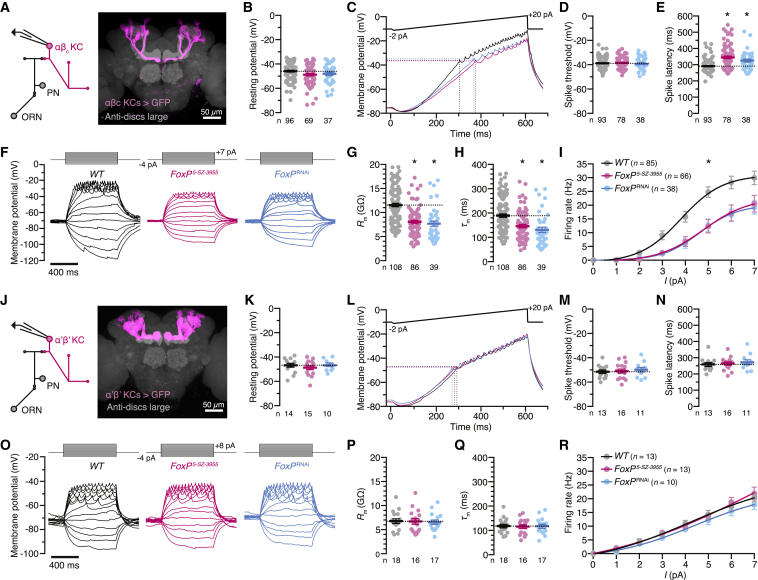
FoxP Determines the Biophysical Properties of αβ_c_ KCs (A) Targeted whole-cell recording from αβ_c_ KCs expressing *NP7175-GAL4*-driven CD8::GFP (magenta). ORN, olfactory receptor neuron; PN, projection neuron. Synaptic structures were counterstained with an antibody against discs large (gray). (B) Resting membrane potentials of αβ_c_ KCs in wild-type flies (black), homozygous *FoxP*^*5-SZ-3955*^ mutants (red), or flies expressing *NP7175-GAL4*-driven *FoxP*^RNAi^ (blue) (circles, individual KCs; bars, means ± SEM). One-way ANOVA failed to detect a significant difference between genotypes (p = 0.0511). (C–E) Voltage responses (C) of αβ_c_ KCs in wild-type flies (black), homozygous *FoxP*^*5-SZ-3955*^ mutants (red), or flies expressing *NP7175-GAL4*-driven *FoxP*^RNAi^ (blue) to ramps of depolarizing current. Cells were held at –75 ± 2 mV at the start of the ramp. FoxP-deficient αβ_c_ KCs initiate action potentials at the same membrane voltage as wild-type cells (D, p = 0.7143) but reach threshold later during the ramp (E, p < 0.0001). Circles, individual KCs; bars, means ± SEM; asterisks, significant differences from wild-type in post hoc comparisons following one-way ANOVA or Kruskal-Wallis test. (F–H) Voltage responses (F) of αβ_c_ KCs in wild-type flies (black), homozygous *FoxP*^*5-SZ-3955*^ mutants (red), or flies expressing *NP7175-GAL4*-driven *FoxP*^RNAi^ (blue) to steps of depolarizing current. FoxP-deficient αβ_c_ KCs have lower input resistances (*R*_m_) (G, p < 0.0001) and shorter membrane time constants (τ_m_) than wild-type cells (H, p < 0.0001). Circles, individual KCs; bars, means ± SEM; asterisks, significant differences from wild-type in post hoc comparisons following Kruskal-Wallis tests. (I) Spike frequencies evoked by depolarizing current injections into αβ_c_ KCs of wild-type flies (black), homozygous *FoxP*^*5-SZ-3955*^ mutants (red), or flies expressing *NP7175-GAL4*-driven *FoxP*^RNAi^ (blue). The cells were held at an initial membrane potential of –70 ± 5 mV, at which spiking is suppressed. Data are means ± SEM. *F* test detected a significant difference between the current-spike frequency functions of wild-type and FoxP-deficient αβ_c_ KCs (p < 0.0001). (J) Targeted whole-cell recording from α’β’ KCs expressing *VT030604-GAL4*-driven CD8::GFP (magenta). ORN, olfactory receptor neuron; PN, projection neuron. Synaptic structures were counterstained with an antibody against discs large (gray). (K) Resting membrane potentials of α’β’ KCs in wild-type flies (black), homozygous *FoxP*^*5-SZ-3955*^ mutants (red), or flies expressing *VT030604-GAL4*-driven *FoxP*^RNAi^ (blue) (circles, individual KCs; bars, means ± SEM). One-way ANOVA failed to detect a significant difference between genotypes (p = 0.6016). (L–N) Voltage responses (L) of α’β’ KCs in wild-type flies (black), homozygous *FoxP*^*5-SZ-3955*^ mutants (red), or flies expressing *VT030604-GAL4*-driven *FoxP*^RNAi^ (blue) to ramps of depolarizing current. FoxP-deficient α’β’ KCs initiate action potentials at the same membrane voltage (M, p = 0.8062) and with the same latency as wild-type cells (N, p = 0.7373). Circles, individual KCs; bars, means ± SEM; asterisks, significant differences from wild-type in post hoc comparisons following one-way ANOVA or Kruskal-Wallis test. (O–Q) Voltage responses (O) of α’β’ KCs in wild-type flies (black), homozygous *FoxP*^*5-SZ-3955*^ mutants (red), or flies expressing *VT030604-GAL4*-driven *FoxP*^RNAi^ (blue) to steps of depolarizing current. FoxP-deficient and wild-type α’β’ KCs have identical input resistances (*R*_m_) (P, p = 0.9497) and membrane time constants (τ_m_) (Q, p = 0.9771). Circles, individual KCs; bars, means ± SEM. (R) Spike frequencies evoked by depolarizing current injections into α’β’ KCs of wild-type flies (black), homozygous *FoxP*^*5-SZ-3955*^ mutants (red), or flies expressing *VT030604-GAL4*-driven *FoxP*^RNAi^ (blue). Data are means ± SEM. *F* test failed to detect a significant difference between the current-spike frequency functions of wild-type and FoxP-deficient α’β’ KCs (p = 0.0660). See also Figure S3.

**Figure S3:**
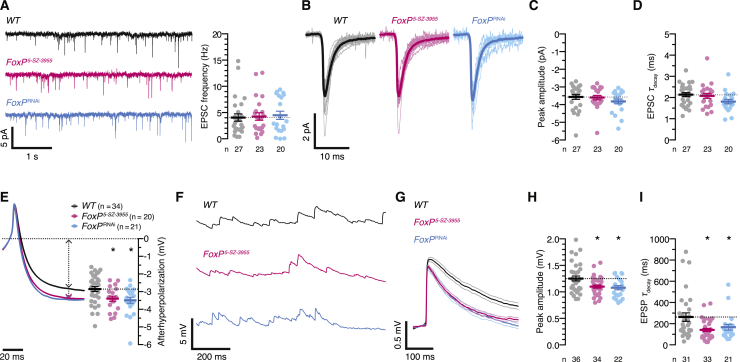
FoxP Does Not Regulate Synaptic Transmission to αβ_c_ KCs, Related to Figure 2 (A) Example transmembrane currents (left, holding potential –90 mV) and mean frequency of spontaneous EPSCs (right) of αβ_c_ KCs in wild-type flies (black), homozygous *FoxP*^*5-SZ-3955*^ mutants (red), or flies expressing *NP7175-GAL4*-driven *FoxP*^RNAi^ (blue). Kruskal-Wallis test failed to detect a significant difference among genotypes (p = 0.8432). Circles, individual KCs; bars, means ± SEM. (B–D) EPSC waveforms at a holding potential of –90 mV (B; shaded and solid lines, cell and population averages) in wild-type flies (black), homozygous *FoxP*^*5-SZ-3955*^ mutants (red), or flies expressing *NP7175-GAL4*-driven *FoxP*^RNAi^ (blue). Cell numbers are indicated in (C). Kruskal-Wallis tests failed to detect significant differences of peak EPSC amplitudes (C, p = 0.2669) or decay time constants (D, p = 0.0507) among genotypes. Circles, individual KCs; bars, means ± SEM. (E) Average action potential waveforms (left) and afterhyperpolarization amplitudes (right) of αβ_c_ KCs in wild-type flies (black), homozygous *FoxP*^*5-SZ-3955*^ mutants (red), or flies expressing *NP7175-GAL4*-driven *FoxP*^RNAi^ (blue). Circles, individual KCs; bars, means ± SEM. Kruskal-Wallis test detected a significant genotype effect (p = 0.0058); asterisks denote significant differences from wild-type in post hoc comparisons. (F) Example membrane potential traces of αβ_c_ KCs in wild-type flies (black), homozygous *FoxP*^*5-SZ-3955*^ mutants (red), or flies expressing *NP7175-GAL4*-driven *FoxP*^RNAi^ (blue). (G–I) EPSP waveforms (E; solid and shaded lines, means ± SEM) in wild-type flies (black), homozygous *FoxP*^*5-SZ-3955*^ mutants (red), or flies expressing *NP7175-GAL4*-driven *FoxP*^RNAi^ (blue). Cell numbers are indicated in (H). FoxP-deficient αβ_c_ KCs have lower mEPSP amplitudes (H, p < 0.0036) and shorter decay time constants (τ_decay_) than wild-type cells (I, p = 0.0299). Circles, individual KCs; bars, means ± SEM; asterisks, significant differences from wild-type in post hoc comparisons following one-way ANOVA or Kruskal-Wallis test. In 5 αβ_c_ KCs of wild-type flies, 1 αβ_c_ KC of a *FoxP*^*5-SZ-3955*^ mutant, and 1 αβ_c_ KC of a *FoxP*^RNAi^ fly, a satisfactory single-exponential fit to the decaying phase of the EPSP could not be found; these cells were excluded from the analyis of τ_decay_ in (I).

Whole-cell patch-clamp recordings *in vivo* (Figure 2A) supported this conclusion. αβ_c_ KCs of wild-type flies, homozygous *FoxP*^*5-SZ-3955*^ mutants, and flies expressing *FoxP*^RNAi^ under *NP7175-GAL4* control rested—the marginal hyperpolarization of FoxP-deficient neurons notwithstanding—at broadly similar membrane potentials (Figure 2B) and received synaptic inputs at comparable basal rates (Figure S3A); these inputs gave rise to the same average excitatory postsynaptic current (EPSC) at a holding potential of –90 mV (Figures S3B–S3D). Regardless of genotype, action potentials were initiated at similar voltages during steps or ramps of depolarizing currents (Figures 2C–2F), but the lower input resistances (Figure 2G) and shorter membrane time constants (Figure 2H) of FoxP-deficient neurons meant that larger currents were required to bring these cells to threshold (Figures 2C and 2F). Because the necessary current amplitudes were reached later during depolarizing ramps (Figure 2C), the spike latencies of FoxP-deficient neurons exceeded those of wild-type cells (Figure 2E).

In contrast to the characteristic quiescence of αβ_c_ KCs at rest, many FoxP-negative α’β’ KCs (Figure 2J) were spontaneously active at similar membrane potential baselines (Figure 2K), reflecting their lower action potential thresholds (Figures 2L–2O). α’β’ KCs had lower input resistances and shorter membrane time constants than αβ_c_ KCs (Figures 2O–2Q) and transduced depolarizing current steps nearly linearly into proportional spike rate increases (Figure 2R), whereas αβ_c_ KCs followed sigmoidal current-spike frequency functions (Figure 2I). As would be expected for FoxP-negative neurons (Figure 1B), none of the biophysical characteristics of α’β’ KCs were perturbed after interference with the expression of FoxP, be it ubiquitously through mutation or in an α’β’ KC-restricted manner through *VT030604-GAL4-*driven *FoxP*^RNAi^ (Figures 2K–2R).

### FoxP Represses Shal in αβ_c_ KCs

**Figure 3:**
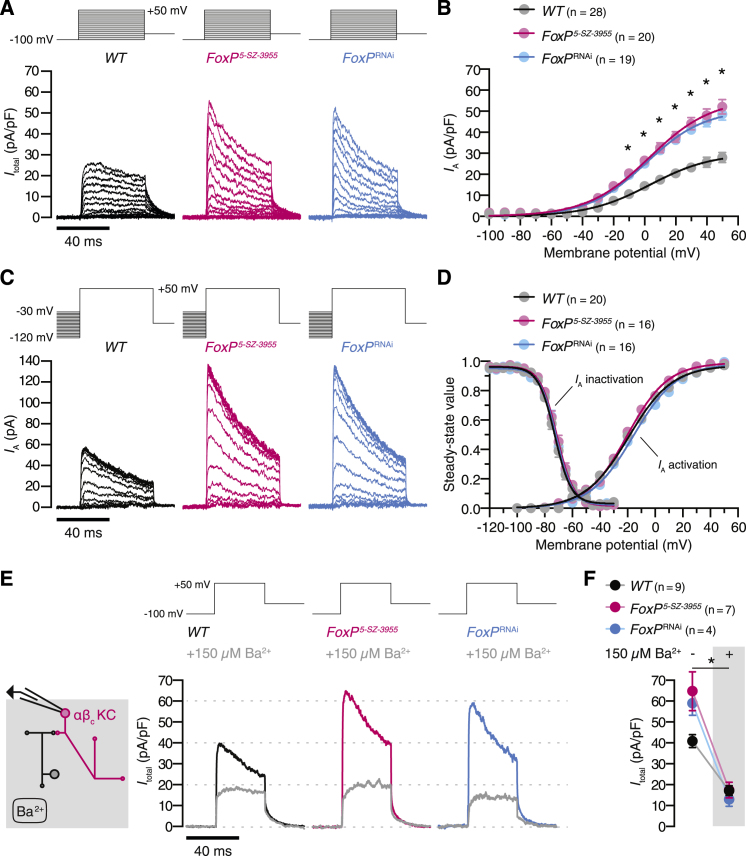
FoxP Regulates a Voltage-Dependent, Ba^2+^-Sensitive Potassium Current of αβ_c_ KCs (A) Potassium current densities, evoked by voltage steps from a holding potential of –100 mV to the indicated probe potentials, in αβ_c_ KCs of wild-type flies (black), homozygous *FoxP*^*5-SZ-3955*^ mutants (red), or flies expressing *NP7175-GAL4*-driven *FoxP*^RNAi^ (blue). (B) A-type potassium current densities in FoxP-deficient αβ_c_ KCs are increased relative to wild-type cells (B, p < 0.0001). Circles, means ± SEM; asterisks, significant differences from wild-type in post hoc comparisons following two-way repeated-measures ANOVA. (C) A-type potassium currents evoked by stepping αβ_c_ KCs of wild-type flies (black), homozygous *FoxP*^*5-SZ-3955*^ mutants (red), or flies expressing *NP7175-GAL4*-driven *FoxP*^RNAi^ (blue) from variable holding potentials (–120 mV to –30 mV) to a probe potential of +50 mV. (D) Steady-state activation and inactivation curves of A-type potassium currents in αβ_c_ KCs of wild-type flies (black), homozygous *FoxP*^*5-SZ-3955*^ mutants (red), or flies expressing *NP7175-GAL4*-driven *FoxP*^RNAi^ (blue). Circles, means ± SEM; solid lines, Boltzmann fits. Kruskal-Wallis tests failed to detect significant genotype effects on half-activation voltage (p = 0.0806), activation slope factor (p = 0.1996), half-inactivation voltage (p = 0.4735), and inactivation slope factor (p = 0.8752). (E) Average potassium current densities in response to a single voltage pulse before (colored lines) and after (gray lines) the bath application of 150 μM Ba^2+^ in αβ_c_ KCs of wild-type flies (black), homozygous *FoxP*^*5-SZ-3955*^ mutants (red), or flies expressing *NP7175-GAL4*-driven *FoxP*^RNAi^ (blue). (F) Average peak current densities before (left) and after (right) Ba^2+^ application. Data are means ± SEM. Two-way repeated-measures ANOVA detected a significant effect of Ba^2+^ treatment (p < 0.0001) and a significant genotype × treatment interaction (p = 0.0012); the asterisk denotes significant differences in peak current densities before and after the addition of Ba^2+^. Kruskal-Wallis tests detected a significant difference in peak current densities among wild-type and FoxP-deficient αβ_c_ KCs before (p = 0.0034), but not after (p = 0.2968), the addition of Ba^2+^. See also Figure S4.

**Figure S4:**
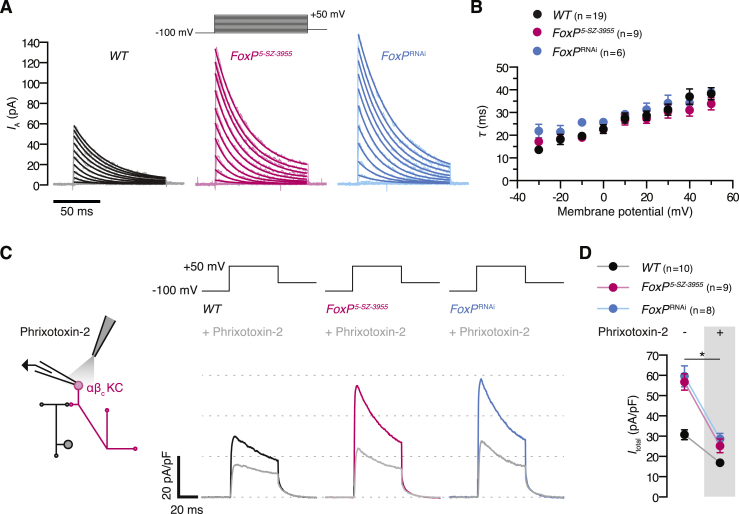
A-Type Potassium Currents of αβ_c_ KCs Show Inactivation Kinetics and Toxin Sensitivities Characteristic of Shal, Related to Figure 3 (A) Exponential fits (solid lines) to average A-type potassium currents (shaded lines) evoked by stepping αβ_c_ KCs of wild-type flies (black), homozygous *FoxP*^*5-SZ-3955*^ mutants (red), or flies expressing *NP7175-GAL4*-driven *FoxP*^RNAi^ (blue) from a holding potential of –100 mV to the indicated probe potentials. (B) Inactivation time constants τ of *I*_A_ in αβ_c_ KCs of wild-type flies (black), homozygous *FoxP*^*5-SZ-3955*^ mutants (red), or flies expressing *NP7175-GAL4*-driven *FoxP*^RNAi^ (blue) as functions of voltage. Two-way repeated-measures ANOVA failed to detect a significant interaction between genotype and the voltage-dependence of τ (p = 0.8943). (C) Pressure application of phrixotoxin-2 to the soma of an αβ_c_ KC. Average potassium current densities in response to a single voltage pulse before (colored traces) and after (gray traces) the application of phrixotoxin-2 to αβ_c_ KCs of wild-type flies (black), homozygous *FoxP*^*5-SZ-3955*^ mutants (red), or flies expressing *NP7175-GAL4*-driven *FoxP*^RNAi^ (blue). (D) Average peak current densities before (left) and after (right) the application of phrixotoxin-2. Data are means ± SEM. Two-way repeated-measures ANOVA detected significant effects of genotype and toxin (p < 0.0001 for both effects) and a significant genotype × toxin interaction (p = 0.0050); the asterisk denotes significant differences in peak current densities before and after the application of phrixotoxin-2.

The identical spike thresholds of wild-type and FoxP-deficient αβ_c_ KCs (Figure 2D) argue against a role of FoxP in regulating voltage-gated sodium or calcium channels, which shape the upstroke of the action potential. Subtle changes in the action potential waveform during repolarization (Figure S3E) and the prolonged latency to spiking during current ramps (Figures 2C and 2E) instead hint at augmented potassium currents in FoxP-deficient αβ_c_ KCs. Voltage-clamp measurements and gene expression analyses confirmed this notion. αβ_c_ KCs expressed a prominent voltage-dependent A-type potassium current (Figures 3A–3C) with half-activation and half-inactivation voltages at steady state of –20.3 ± 1.6 mV and –72.9 ± 1.2 mV, respectively (Figures 3C and 3D), and an inactivation time constant of 37.2 ± 2.4 ms at +50 mV (Figures S4A and S4B). The loss of FoxP nearly doubled the membrane density of this current (Figures 3A–3C) while leaving unchanged its steady-state activation and inactivation curves (Figure 3D). FoxP thus appears to control the cell surface density of the pore-forming α subunit of a voltage-gated potassium (K_V_) channel and not the level of an accessory protein that influences the channel’s gating.

**Figure 4:**
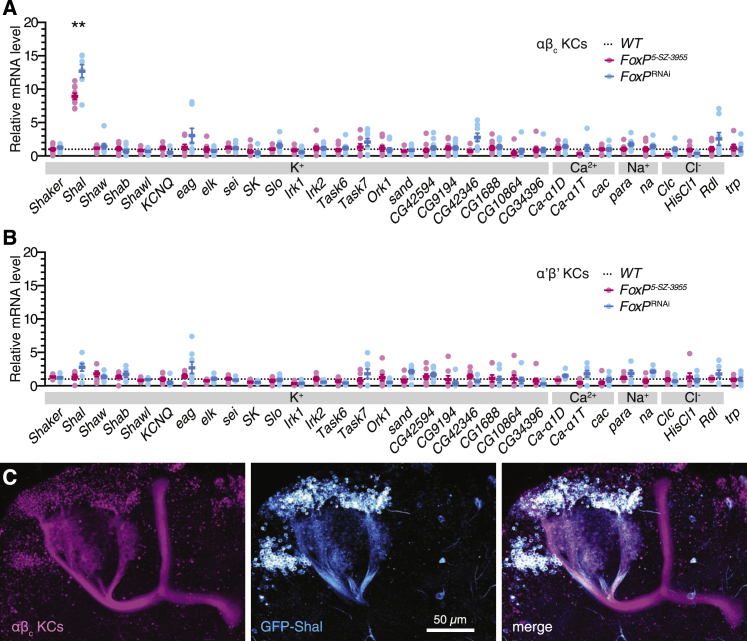
FoxP Represses the Dendritic K_V_ Channel Shal in αβ_c_ KCs (A and B) Levels of polysome-bound ion channel transcripts in αβ_c_ (A) or α’β’ KCs (B) of homozygous *FoxP*^*5-SZ-3955*^ mutants (red) and flies expressing *NP7175-GAL4*- or *VT030604-GAL4*-driven *FoxP*^RNAi^ (blue), relative to corresponding transcript levels in αβ_c_ or α’β’ KCs of wild-type flies (circles, biological replicates; bars, means ± SEM; see Table S1 for sample sizes). Asterisks denote significant differences from wild-type in post hoc comparisons following one-way ANOVA (Bonferroni-corrected p < 0.0016). (C) *NP6024-GAL4*-driven expression of membrane-bound mCherry (magenta) and GFP-tagged Shal (cyan) in αβ_c_ KCs. See also Figure S5 and Tables S1 and S5.

Quantitation of polysome-bound transcripts after cell-specific TRAP (Heiman et al., 2008) identified the K_V_4 channel Shal (Butler et al., 1989) as the sole FoxP target among the pore-forming subunits of 32 potassium, sodium, calcium, and chloride channels encoded in the fly genome (Figure 4A). Shal’s gating parameters (Gasque et al., 2005, Podlaski et al., 2017, Wei et al., 1990) and sensitivity to phrixotoxin-2 (Gasque et al., 2005) and 150 μM Ba^2+^ (Gasparini et al., 2007, Norris and Nerbonne, 2010) matched, despite the distortions inherent in voltage-clamp measurements from non-spherical cells (Rall and Segev, 1985), those of the A-type current expressed by αβ_c_ KCs (Figures 3D–3F and S4). Mutation of the *FoxP* locus or RNAi-mediated depletion of FoxP elevated the amount of polysome-bound *Shal* mRNA in αβ_c_ KCs ∼10-fold; the levels of all other ion channel transcripts remained unaltered (Figure 4A). Global or restricted interference with the function of FoxP had no significant impact on ion channel gene expression in α’β’ KCs (Figure 4B).

**Figure S5:**
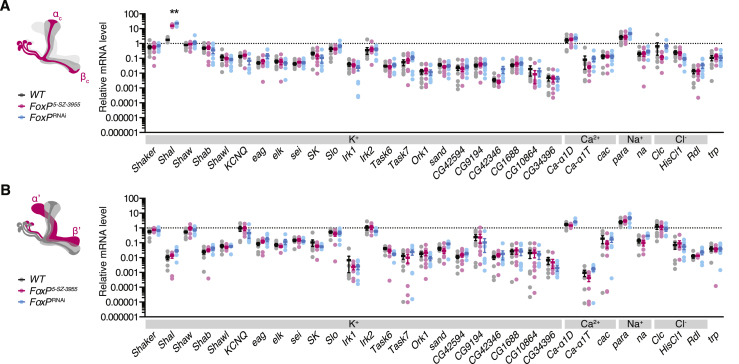
FoxP Represses Shal in αβ_c_ KCs, Related to Figure 4 (A and B) Levels of polysome-bound ion channel transcripts in αβ_c_ KCs (A) or α’β’ KCs (B) of wild-type flies (black), homozygous *FoxP*^*5-SZ-3955*^ mutants (red), or flies expressing *FoxP*^RNAi^ (blue), relative to the geometric mean of three marker gene transcripts (*Gpdh*, *Tbp*, and *Ef1α100E*; circles, biological replicates; bars, means ± SEM; see Table S1 for sample sizes). Note the logarithmic scale. One-way ANOVA detected a significant genotype effect on *Shal* levels in αβ_c_ KCs (p < 0.0001); asterisks denote significant differences from wild-type in post hoc comparisons.

The channel repertoires of αβ_c_ and α’β’ KCs were dominated by different K_V_ types. While *Shal* represented the most abundant K_V_ channel mRNA species in αβ_c_ KCs (Figure S5A), *Shaker* and *Shaw* predominated in α’β’ KCs (Figure S5B). Distinct mechanisms thus operate in different KC classes to control the expression of Shal: in αβ_c_ KCs, which contain both Shal and FoxP (Figures 1B and S5A), the repressive action of FoxP limits the dosage of *Shal* mRNA, whereas in α’β’ neurons, which express little Shal and no FoxP (Figures 1B and S5B), a FoxP-independent mechanism inhibits transcription of the *Shal* locus. As is evident from the disparity between the increase in *Shal* transcript levels (∼10-fold; Figure 4A) and Shal currents (∼2-fold; Figure 3B) in FoxP-deficient αβ_c_ KCs, an additional layer of post-transcriptional control also exists.

### FoxP, Shal, and Synaptic Integration

**Figure 5:**
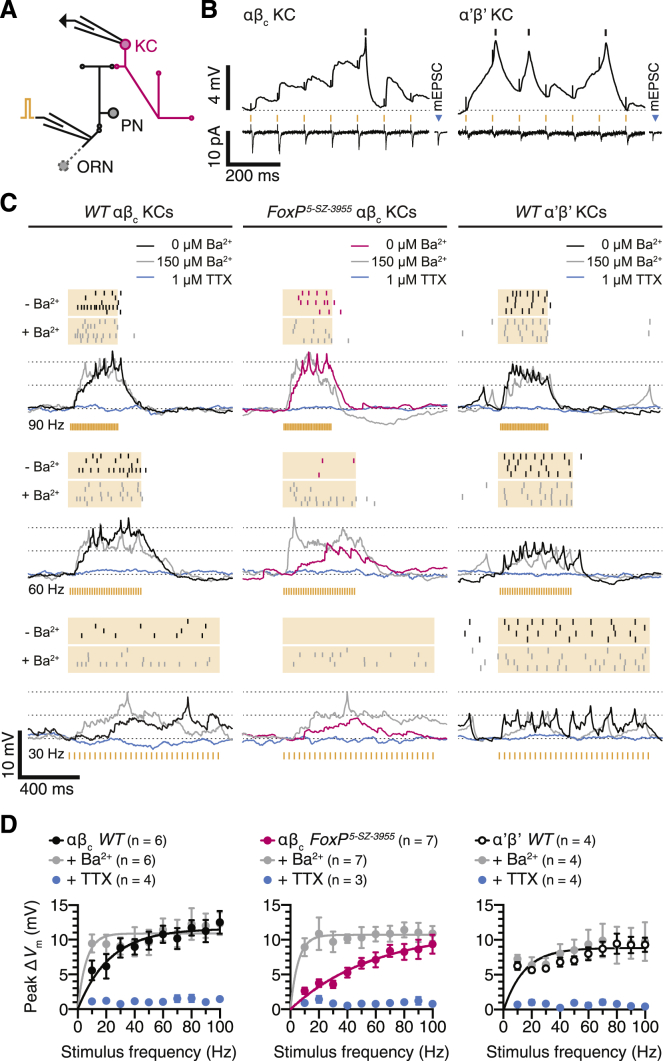
FoxP Tunes the Integrative Properties of αβ_c_ KCs (A) Antennal nerve stimulation and targeted whole-cell recording from KCs expressing *NP7175-GAL4*- or *VT030604-GAL4*-driven CD8::GFP. ORN, olfactory receptor neuron; PN, projection neuron. (B) Sequentially recorded voltage (top) and transmembrane current responses (bottom, holding potential –70 mV) of the same αβ_c_ (left) or α’β’ KC (right) in a wild-type fly during antennal nerve stimulation at 10 Hz (orange marks); the averages of 119 (left) and 17 (right) miniature EPSCs recorded in the same cells are shown for comparison (mEPSC). (C) Examples of spike rasters (top) and voltage responses (bottom) of 5 αβ_c_ KCs in wild-type flies (left) and homozygous *FoxP*^*5-SZ-3955*^ mutants (center) and of 4 α’β’ KCs in wild-type flies (right) during antennal nerve stimulation at the indicated frequencies, in control conditions (black or red) and after the sequential addition of 150 μM Ba^2+^ to block Shal (gray) and 1 μM tetrodotoxin (TTX) to block action potentials (blue). Each row in the rasters depicts a different KC. Stimulus artifacts were removed for clarity. (D) Stimulus-response curves of αβ_c_ KCs in wild-type flies (left) and homozygous *FoxP*^*5-SZ-3955*^ mutants (center) and of α’β’ KCs in wild-type flies (right) during antennal nerve stimulation in control conditions (black or red) and after the sequential addition of 150 μM Ba^2+^ to block Shal (gray) and 1 μM TTX to block action potentials (blue). Data are means ± SEM. *F* tests detected significant effects of 150 μM Ba^2+^ on the stimulus-response curves of αβ_c_ KCs in wild-type flies (p = 0.0288) and homozygous *FoxP*^*5-SZ-3955*^ mutants (p < 0.0001), but not of α’β’ KCs in wild-type flies (p = 0.4186). See also Figure S6.

**Figure S6:**
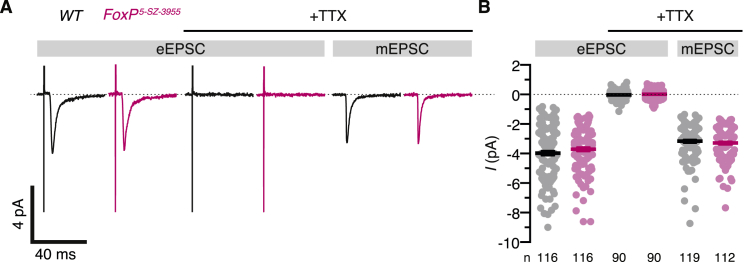
EPSCs Evoked in αβ_c_ KCs by Antennal Nerve Stimulation: Comparison with Miniature EPSCs, Related to Figure 5 (A) Average transmembrane currents (holding potential –90 mV) of αβ_c_ KCs in wild-type flies (black) or homozygous *FoxP*^*5-SZ-3955*^ mutants (red). EPSCs evoked by 50 μs voltage pulses (left) were blocked by 1 μM tetrodotoxin (TTX; center); to minimize the fraction of transmission failures, stimulation voltages exceeded those at which EPSCs were first detected by ∼25%. The peak currents of spontaneously occurring miniature EPSCs in 1 μM TTX (mEPSCs, right) averaged 80–89% of those of eEPSCs. (B) Peak eEPSC currents in the absence and presence of 1 μM TTX (left and center) versus peak mEPSC currents in 1 μM TTX (right), in αβ_c_ KCs of wild-type flies (black) and homozygous *FoxP*^*5-SZ-3955*^ mutants (red). Circles, individual EPSCs; bars, means ± SEM. Two-way ANOVA detected a significant effect of release mode (evoked versus spontaneous, p < 0.0001) but not of genotype (p = 0.6094).

The somatodendritic location (Diao et al., 2010) (Figure 4C) and hyperpolarized voltage operating range of Shal (Figure 3D) suggest that the channel, like its mammalian ortholog K_V_4.2 in hippocampal pyramidal cells (Cai et al., 2004, Hoffman et al., 1997, Kim et al., 2005, Sheng et al., 1992), dampens dendritic voltage deflections and thereby regulates the amplitude, time course, and propagation of excitatory postsynaptic potentials (EPSPs). To test for a role of Shal (and, indirectly, of FoxP) in synaptic integration, we recorded from αβ_c_ and α’β’ KCs *in vivo* while electrically stimulating 30 synaptic inputs from olfactory projection neurons at different frequencies (Figures 5A, 5B, and S6). The majority of evoked events (∼80%) were unitary, as the average synaptic current exceeded the average miniature EPSC by 12%–25% (Figure S6). During stimulus trains, the membrane potentials of αβ_c_ KCs in wild-type flies climbed in a stepwise fashion from resting potential to spike threshold, bridging the average potential difference of 8.2 mV by integrating 4–30 synaptic quanta (Figures 5B and 5C). Inputs were summed most efficiently when delivered at high frequencies (Figures 5C and 5D), but even EPSPs spaced 50 or even 100 ms apart could add up sufficiently to drive spiking (Figure 5B). By contrast, just one or two EPSPs could close the narrow voltage gap between the resting potentials and spike thresholds of α’β’ KCs, obviating the need for extensive synaptic integration (Figures 5B–5D).

The EPSC amplitudes of FoxP-deficient and wild-type αβ_c_ KCs were identical when the cells were voltage-clamped below the activation threshold of Shal (Figures S3B and S3C). In current-clamp recordings near resting potential, however, spontaneous EPSPs in αβ_c_ KCs of *FoxP* mutants were smaller and decayed faster (Figures S3G–S3I), and evoked EPSPs were dissipated more readily (Figure 5C), than those in wild-type cells. As a consequence, opportunities for temporal summation of synaptic inputs were curtailed, and only high-frequency stimulation could produce cumulative membrane depolarizations that breached action potential threshold (Figures 5C and 5D). The addition of 150 μM Ba^2+^, which at this concentration selectively blocks K_V_4 channels (Gasparini et al., 2007, Norris and Nerbonne, 2010) (Figures 3E and 3F), steepened the stimulus-response curves of wild-type and FoxP-deficient cells to different degrees (reflecting the different amounts of Shal expressed by these neurons) and brought them into precise alignment (p = 0.9702, *F* test; Figure 5D): αβ_c_ KCs of both wild-type and mutant flies now required just a handful of synaptic impulses to emit action potentials. The lack of a Ba^2+^ effect on α’β’ KCs, which express high levels of *Shaker* and *Shaw* but little, if any, *Shal* (Figure S5B), attests to the specificity of the pharmacological block (Figures 5C and 5D).

### FoxP, Shal, and Reaction Times

**Figure 6:**
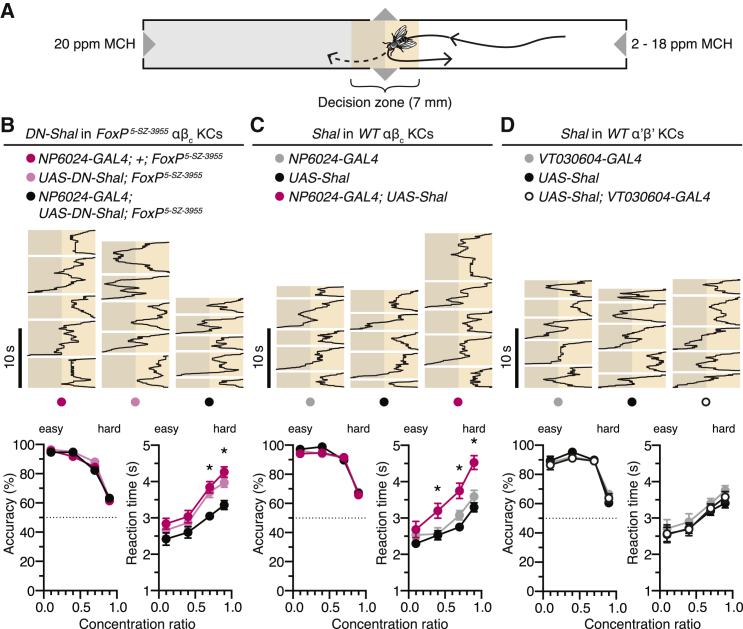
Shal Currents in αβ_c_ KCs Determine Reaction Times (A) Measurement of reaction times and decision accuracies. Two odor streams converge in a 7-mm-wide decision zone (orange) at the center of a narrow chamber. Flies are trained to avoid 20 ppm of 4-methylcyclohexanol (MCH, gray) and must discriminate the reinforced from a lower MCH concentration (2–18 ppm); the concentration ratio during testing determines the difficulty of the task. The time spent in the decision zone is quantified as the reaction time. (B–D) Example traces (top) of position on the long chamber axis versus time of flies of the indicated genotypes in the decision zone: homozygous *FoxP*^*5-SZ-3955*^ mutants expressing dominant-negative Shal in αβ_c_ KCs (B), wild-type flies overexpressing functional Shal in αβ_c_ KCs (C), or wild-type flies overexpressing functional Shal in α'β' KCs (D), along with their respective parental controls; the MCH concentration ratio is 0.9. Accuracy (bottom left) and reaction time (bottom right) of flies of the indicated genotypes as functions of the MCH concentration ratio. Data are means ± SEM (see Table S2 for sample sizes); asterisks denote significant differences of experimental flies from both parental controls (accuracy, Dunn’s test following one-way Kruskal-Wallis test; reaction time, Kolmogorov-Smirnov test with Bonferroni-corrected p < 0.00625). See also Table S2.

The ability to erase the biophysical difference between *FoxP* genotypes simply by blocking Shal (Figures 5C and 5D) reaffirms that this ion channel is a crucial mediator of FoxP’s effects on αβ_c_ KCs. We therefore examined whether an analogous manipulation could also correct the reaction time phenotype of *FoxP* mutants. Flies were trained to avoid a specific concentration of 4-methylcyclohexanol (MCH) by pairing odor exposures with electric foot shock. The animals then had to discriminate the reinforced MCH concentration (20 ppm) from a lower intensity (2–18 ppm) of the same odor (Figure 6A). The difficulty of discrimination was adjusted by varying the MCH concentration ratio during testing. Control flies make rapid, accurate decisions in easy discriminations (concentration ratio 0.1) and slow, error-prone choices in difficult tasks (concentration ratio 0.9) (DasGupta et al., 2014). Homozygous carriage of the *FoxP*^*5-SZ-3955*^ mutation exacted a steeper difficulty-dependent cost of decision time (DasGupta et al., 2014), but the chronometric function was restored to its wild-type shape when *FoxP* mutants expressed a transgene (*UAS-DN-Shal*) encoding a dominant-negative Shal subunit (Ping et al., 2011) selectively in αβ_c_ KCs (Figure 6B). The temperature-inducible overexpression of functional Shal in αβ_c_ KCs (Figure 6C), but not in α’β’ KCs (Figure 6D), of wild-type flies had the opposite effect; it recapitulated the *FoxP* mutant phenotype. Bidirectional tuning of Shal currents in αβ_c_ KCs thus produced a spectrum of wild-type and mutant reaction times, independently of the genotype of the *FoxP* locus (Figures 6B and 6C). These data establish αβ_c_ KCs as the decision-relevant site, and repression of Shal as the decision-relevant mechanism, of FoxP’s action.

### Neurometric Functions Based on First Spikes

**Figure 7:**
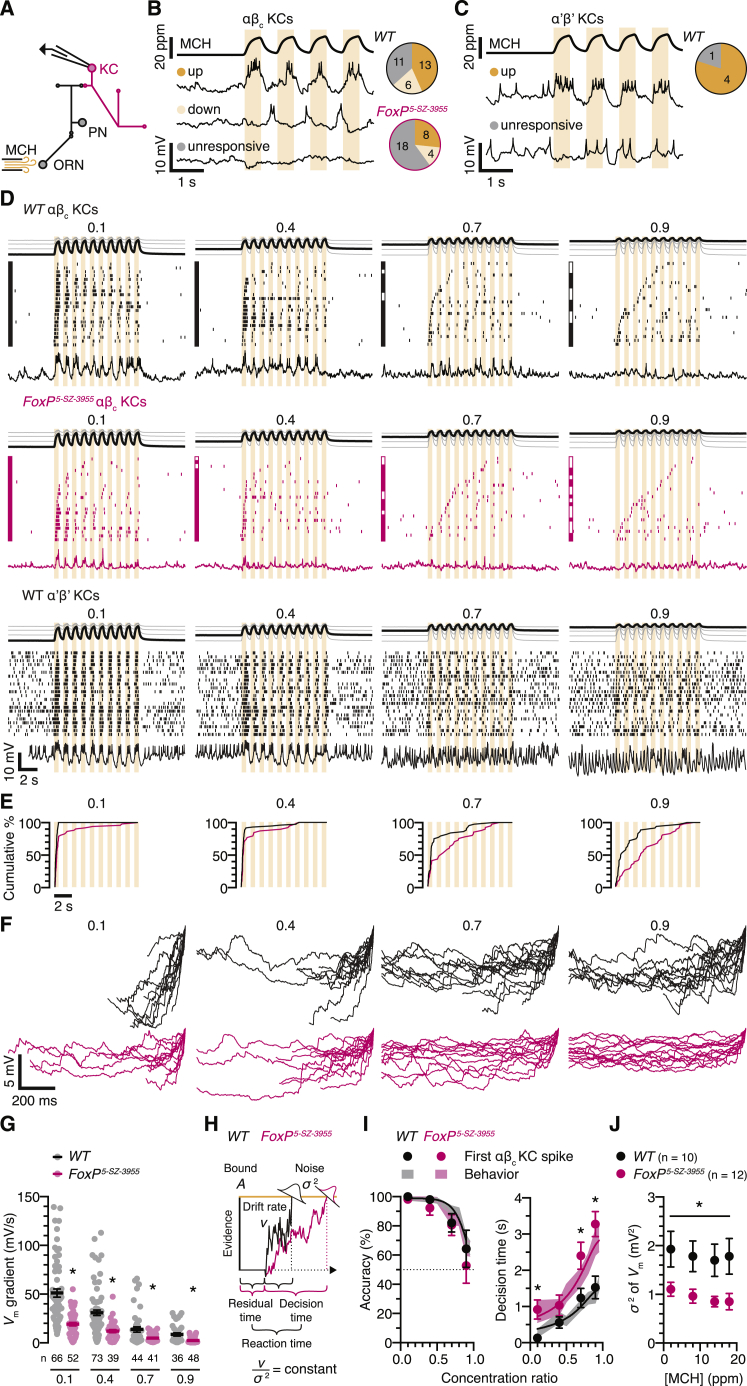
The First Odor-Evoked αβ_c_ KC Spike Predicts Behavior (A) Odor stimulation and targeted whole-cell recording from KCs expressing *NP7175-GAL4*- or *VT030604-GAL4*-driven CD8::GFP. ORN, olfactory receptor neuron; PN, projection neuron; MCH, 4-methylcyclohexanol. (B and C) MCH concentrations (top) and membrane voltages (bottom) of up (orange), down (pale orange), and unresponsive (gray) αβ_c_ (B) or α’β’ KCs (C) during repeated steps between base (2 ppm) and peak MCH concentrations (20 ppm); pie charts indicate the proportions of functional KC classes encountered in wild-type flies and homozygous *FoxP*^*5-SZ-3955*^ mutants. (D) Examples of spike rasters and voltage responses of up αβ_c_ KCs in wild-type flies (black, top) and homozygous *FoxP*^*5-SZ-3955*^ mutants (red) and of α’β’ KCs in wild-type flies (black, bottom) during ten odor intensity cycles between a variable base (2–18 ppm) and a constant peak (20 ppm) MCH concentration at 1 Hz. Measured MCH concentration time courses at the different base-to-peak ratios are displayed on top. The spike rasters are sorted, in ascending order from the bottom, by the latency of the first spike. Filled and open squares in the margins mark trials whose first spikes occur after odor concentration changes in the preferred or null (“correct” or “incorrect”) directions (Figure S7B). (E) Cumulative frequency distributions of spike latencies after stimulus onset of up αβ_c_ KCs in wild-type flies (black) and homozygous *FoxP*^*5-SZ-3955*^ mutants (red) at the MCH concentration ratios indicated on top. (F) Examples of membrane voltages preceding the first αβ_c_ KC spike after stimulus onset in wild-type flies (black) and homozygous *FoxP*^*5-SZ-3955*^ mutants (red), at the MCH concentration ratios indicated on top. The traces are aligned to the upstroke of the action potential (peak of the first derivative) and depict a period of ≤ 1 s. (G) Voltage gradients from stimulus onset to the upstroke of the first αβ_c_ KC spike in wild-type (black) and *FoxP*^*5-SZ-3955*^ mutant flies (red) at the indicated MCH concentration ratios. Circles, individual trials; bars, means ± SEM. Two-way repeated-measures ANOVA detected significant effects of odor contrast (p < 0.0001) and genotype (p < 0.0001) and a significant genotype × contrast interaction (p < 0.0001); asterisks denote significant differences between genotypes. (H) Drift-diffusion model of evidence accumulation. Homozygous *FoxP*^*5-SZ-3955*^ mutants (red) exhibit lower drift rate (*v*) and noise (σ^2^) than do wild-type flies (black). (I) Neurometric predictions of accuracy (left) and decision time (right; decision time = reaction time – residual time) as functions of MCH concentration ratio. The predictions are based on the timing and fidelity of the first MCH-evoked up αβ_c_ KC spikes in wild-type flies (black) and homozygous *FoxP*^*5-SZ-3955*^ mutants (red). Data are means ± SEM (see Table S3 for sample sizes); asterisks, significant differences from wild-type (accuracy, Mann-Whitney U test; reaction time, Kolmogorov-Smirnov test; both with Bonferroni-corrected p < 0.0125). Shaded bands represent 95% confidence intervals of accuracy and decision time measurements; solid lines depict the fit of a drift-diffusion model to these behavioral data. (J) Average membrane potential variances of αβ_c_ KCs in wild-type flies (black) and homozygous *FoxP*^*5-SZ-3955*^ mutants (red) as functions of base MCH concentration. Data are means ± SEM. Two-way ANOVA detected a significant genotype effect (p < 0.0164). See also Figure S7 and Tables S3 and S4.

The membrane potential responses of αβ_c_ KCs to changes in odor concentration, intended to emulate a fly’s experience of the decision zone in a behavioral chamber (Claridge-Chang et al., 2009, DasGupta et al., 2014), offer a bridge between biophysics and behavior (Figure 7). A continuous stream of MCH was stepped repeatedly between base (2–18 ppm, variable between trials) and peak concentrations (20 ppm, constant across trials) (Figures 7A–7D), recreating the exact range of odor intensities and intensity contrasts encountered during behavioral testing (Figure 6). The 500-ms intervals between odor intensity changes (Figures 7B–7D) approximated the times flies spend on one side of the decision zone (Claridge-Chang et al., 2009, DasGupta et al., 2014), whereas periodic stimulation mimicked their back-and-forth movements and allowed us to quantify αβ_c_ KC responses to concentration steps in the preferred and null directions (see below), a prerequisite for neurometric estimates of accuracy (Britten et al., 1992).

**Figure S7:**
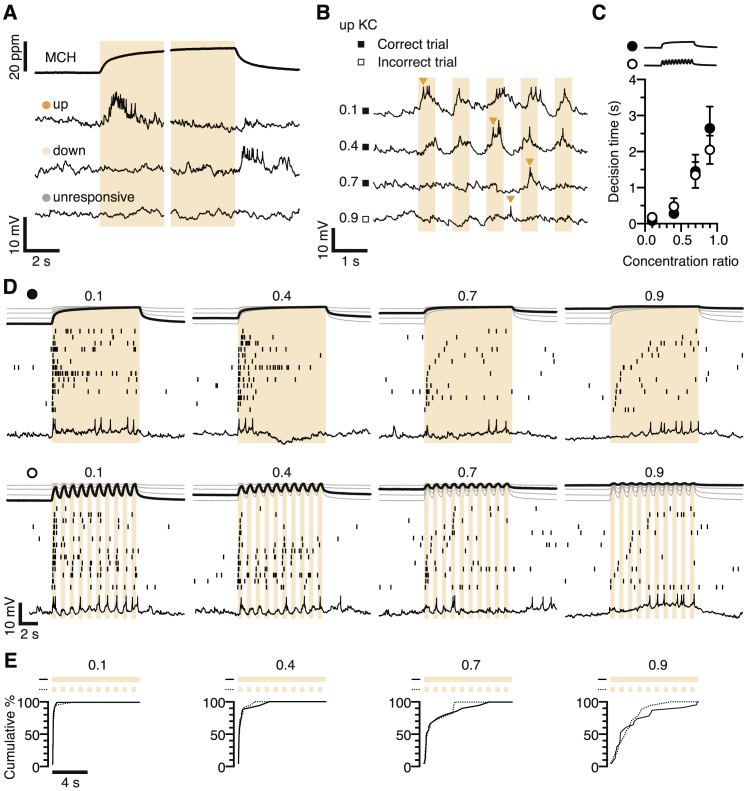
Average Spike Latencies of αβ_c_ KCs Are Independent of Odor Stimulus Waveform, Related to Figure 7 (A) MCH concentrations (top) and membrane voltages (bottom) of up (orange), down (pale orange), and unresponsive αβ_c_ KCs (gray) in wild-type flies during a single 10 s step between base (2 ppm) and peak MCH concentrations (20 ppm) and back. (B) Voltage responses of an up αβ_c_ KC during 10 odor intensity cycles between a variable base (2–18 ppm) and a constant peak (20 ppm) MCH concentration. Only the first five of the 10 odor stimulation cycles are shown. MCH base-to-peak ratios are indicated on the left; filled and open squares mark trials whose first spikes (arrowheads) occur after odor concentration changes in the preferred or null (“correct” or “incorrect”) directions. (C) Neurometric predictions of decision times (decision time = reaction time – residual time) as a function of MCH concentration ratio. The predictions are based on the timing of the first MCH-evoked spike in response to a single 10 s concentration step (filled circles) or 10 odor intensity cycles at 1 Hz (open circles). Kolmogorov-Smirnov tests with Bonferroni-corrected p < 0.0125 failed to detect a significant difference between stimulation protocols (p = 0.7559). Data are means ± SEM (see Table S4 for sample sizes). (D) MCH stimulus waveforms, examples of spike rasters, and voltage responses during a single 10 s odor intensity step (top) or 10 odor intensity cycles at 1 Hz (bottom) between a variable base (2–18 ppm) and a constant peak (20 ppm) MCH concentration and back. Measured MCH concentration time courses at the different base-to-peak ratios are displayed on top. The spike rasters are sorted, in ascending order from the bottom, by the latency of the first spike. (E) Cumulative frequency distributions of spike latencies for a single 10 s concentration step (solid lines) and 10 odor intensity cycles at 1 Hz (dashed lines), at the MCH concentration ratios indicated on top.

Current-clamp recordings during blocks of ten stimulation cycles revealed three functional classes of αβ_c_ KC: neurons responding to increases or decreases in MCH concentration (“up” and “down” cells) and unresponsive neurons (Ito et al., 2008) (Figures 7B and S7A). Decisions could thus be based on comparisons of activity in two opponent pools of αβ_c_ KCs representing evidence for the alternative choices, an arrangement reminiscent of the hypothetical “neuron-antineuron” configuration in visual motion discrimination (Britten et al., 1992). Because we found twice as many up as down αβ_c_ KCs (Figure 7B), our subsequent analyses concentrated on them.

In contrast to α’β’ KCs (Figures 7C and 7D), αβ_c_ KCs showed virtually no background spiking activity, regardless of base MCH concentration, and instead responded to concentration changes (Figures 7B, 7D, and S7). In wild-type flies, large fractional increases in odor intensity (from 2 to 20 ppm MCH, a concentration ratio of 0.1) evoked action potentials with short latencies (< 300 ms) that were preceded by steep membrane depolarizations (Figures 7F and 7G) and tended to repeat during the correct phase of many, if not all, successive odor concentration cycles in a block (Figure 7D). Reducing the intensity contrast (by elevating the MCH base) produced shallower, meandering voltage changes (Figures 7F and 7G) that eroded the close temporal coupling between stimulus and spiking response until, at a concentration ratio of 0.9, a median of three stimulation cycles were needed to unlock the first spike (Figure 7D). This up cell spike now often occurred during the incorrect—that is, downward—phase of the concentration cycle (Figures 7D and S7B). Neuronal sensitivity and selectivity, quantified as the timing and fidelity of the first odor-evoked spike, thus declined with diminishing odor contrast. The decline in sensitivity was precipitous in FoxP-deficient αβ_c_ KCs, which suffered excessive spike delays (Figure 7D) or response failures (Figure 7B) that likely reflect the synaptic integration defect exposed during electrical stimulation of the antennal nerve (Figures 5C and 5D).

Altogether, the changes in αβ_c_ KC responses as a function of stimulus contrast, and the influence of FoxP on them, parallel changes in the speed and accuracy of the decision-making animal in perceptual tasks of varying difficulty. To make this notion rigorous, we compared the psycho- and chronometric functions measured behaviorally (DasGupta et al., 2014) with neurometric counterparts constructed from the separately recorded αβ_c_ KC responses. Because the temporal structure of the odor stimulus used to elicit these responses was an imperfect match to the many possible sequences of odor concentration changes experienced by flies sampling the decision zone (Claridge-Chang et al., 2009, DasGupta et al., 2014) (Figure 6), we verified that the exact stimulus waveform had no significant influence on the average spike latency of αβ_c_ KCs (Figures S7C–S7E). The conclusions that follow are therefore robust.

Behavioral performance is well described (DasGupta et al., 2014) by a drift-diffusion model with three free parameters (Bogacz et al., 2006, Ratcliff, 1978, Ratcliff and McKoon, 2008) (Figure 7H). The model splits the reaction time into two additive components: a difficulty- and genotype-dependent decision time and a constant residual time, which is taken up by processes unrelated to the decision itself (Bogacz et al., 2006, Ratcliff, 1978, Ratcliff and McKoon, 2008). In our assay, the lion’s share of the estimated residual time of 1.296 s is spent on locomotion: a fly walking at a measured mean velocity of 2.98 ± 0.08 mm/s in the direction of the long chamber axis will need 1.356 s to travel the average distance of 2.02 ± 0.02 mm from the edge of the decision zone to the empirically determined turning point and back (means ± SEM, n = 775 decisions). After stripping away this behaviorally constrained residual time from the measured reaction times, the mean latency of the first αβ_c_ KC spike predicted the pure decision times of both genotypes at all difficulty levels (Figure 7I). Similarly good fits to the empirical decision accuracies (which are identical for wild-type and *FoxP*^*5-SZ-3955*^ mutant flies) were obtained by tallying the percentages of first spikes released after odor concentration steps in the preferred or null directions (Figure 7I).

The fact that the psychometric functions of wild-type and *FoxP*^*5-SZ-3955*^ mutant flies overlapped while their chronometric functions differed (Figure 7I) places an additional constraint on putative neural mechanisms of sequential sampling. In the drift-diffusion framework (Bogacz et al., 2006, Ratcliff, 1978, Ratcliff and McKoon, 2008), any change that slows the sensory evidence-driven drift of a decision variable toward threshold will not only extend the average reaction time but simultaneously reduce accuracy, due to the relatively augmented influence of diffusion noise σ^2^. When the decision criterion is fixed, as is the spike threshold of wild-type and mutant αβ_c_ KCs relative to resting potential (Figures 2B and 2D), maintaining constant accuracy despite lower drift rate *v* requires a commensurate reduction in noise, keeping *v*/σ^2^ constant (Figure 7H). Such a compensatory change was evident in the subthreshold membrane potential fluctuations of FoxP-deficient αβ_c_ KCs (Figures 7D, 7F, and 7J). Because K_V_4 channels act like shock absorbers that limit large, rapid voltage changes (Cai et al., 2004, Hoffman et al., 1997), their abundance in mutants reduced the average membrane potential variance (σ^2^) to 0.5242 times that of wild-type cells (Figure 7J). Estimates, from reaction time data (DasGupta et al., 2014), of the drift rates (*v*) of homozygous *FoxP*^*5-SZ-3955*^ mutant and wild-type flies yielded a virtually identical ratio of 0.5296, ensuring the required constancy of *v*/σ^2^ across genotypes.

## Discussion

Searches for the mechanisms that allow neurons to accumulate information during decision-making, compare the accumulated signal to a response criterion, and release behavior when the criterion is met have almost exclusively centered on suprathreshold dynamics, which are visible to extracellular electrodes or Ca^2+^ reporters (Hanes and Schall, 1996, Harvey et al., 2012, Latimer et al., 2015, Roitman and Shadlen, 2002, Shadlen and Newsome, 2001). In the prevalent view, ramp-like changes in mean firing rate represent accumulating evidence in the lead-up to a choice (Gold and Shadlen, 2007, Shadlen and Kiani, 2013). Our findings suggest that αβ_c_ KCs integrate subthreshold depolarizations evoked by sequentially arriving quanta of sensory information, and that the ability to execute this integration operation dictates behavioral performance. In contrast to ramping (Hanes and Schall, 1996, Roitman and Shadlen, 2002, Shadlen and Newsome, 2001), stepping (Latimer et al., 2015), or high-dimensional (Harvey et al., 2012) patterns of spiking activity, which must be decoded by still unspecified thresholding mechanisms, synaptic integration reaches a natural endpoint: the discharge of an action potential. It can therefore provide all computational elements needed to map a process of bounded evidence accumulation onto a neuronal medium.

While the ability to combine synaptic inputs is, of course, common to all neurons, αβ_c_ KCs appear to tailor, through a particular constellation of biophysical properties, this generic process to the demands of sequential sampling. A large voltage difference (equal to the linear sum of six or seven coincident EPSPs) separates resting potential and spike threshold (Figures 2B, 2D, and S3H); this makes action potentials infrequent and, when they occur, contingent on the summation of multiple sensory inputs (Figures 5C and 7D–7F). A long membrane time constant (Figure 2H) provides accumulator memory (Figures 5B and 5C), whereas Shal currents may improve accuracy by reducing noise (Figure 7J) and discounting sporadic sensory events (Figures 5C and 5D).

The accord between the behaviorally inferred noise in the decision variable (Figure 7H) and the measured voltage noise of αβ_c_ KCs (Figure 7J) indicates that the membrane potential of these neurons encodes a quantity closely related to a decision variable. Consistent with this view, decisions take the same average time to form as the membrane potential does to reach spike threshold (Figure 7I), and manipulations that advance or delay the first αβ_c_ KC spike have corresponding consequences for reaction times (Figures 6B and 6C). Although the average latency of the first αβ_c_ KC spike can account for the average decision time, this agreement alone cannot establish that αβ_c_ KC are the only integrators along the sensorimotor pathway: the argument naturally hinges on correct decision time estimates by our drift-diffusion model and could be strengthened by extending the analysis from statistical averages to individual trials. However, this will require new methods for measuring and perturbing the membrane potential while decisions are being made.

The existence of up and down cells (Figures 7B and S7A) suggests that a decision entails a race between two integrators, one that accumulates evidence for an increase in odor concentration and another that does the opposite (Shadlen and Kiani, 2013). It is reasonable to assume (though difficult at present to prove) that the winner of the race informs the animal’s choice by activating inherently valued mushroom body output neurons (MBONs) (Aso et al., 2014b). KC-to-MBON synapses depress (Hige et al., 2015, Séjourné et al., 2011) when odor-driven KC and MBON activity coincides with local dopaminergic reinforcement (Aso et al., 2014a, Burke et al., 2012, Claridge-Chang et al., 2009, Liu et al., 2012, Schwaerzel et al., 2003). Because MBONs and reinforcing dopaminergic neurons are twinned in sign-reversed pairs (MBONs directing attraction with aversively reinforcing dopaminergic neurons and vice versa) (Aso et al., 2014a, Aso et al., 2014b), synaptic depression will differentially weaken the connections of the opponent αβ_c_ KC pools with MBONs of opposite valence. In our training regime, the expected net effect of these changes is that activity in up and down cells elicits learned avoidance and approach, respectively. Correct and erroneous choices follow logically from this model: if up cells spike before down cells after an increase in odor intensity, a correct reversal will result; if down cells spike before up cells, an incorrect approach to the negatively reinforced odor will be made. Competition between these “neuron-antineuron” pools (Britten et al., 1992) or the MBONs sampling them could be enhanced by feedforward or recurrent inhibition mediated by GABAergic or glutamatergic MBONs interconnecting mushroom body compartments (Aso et al., 2014a), or by the GABAergic anterior paired lateral neuron (Lin et al., 2014, Papadopoulou et al., 2011). These circuit architectures are similar in essence (Bogacz et al., 2006) to connectionist (Shadlen and Newsome, 2001, Shadlen and Kiani, 2013, Usher and McClelland, 2001) or reduced network models (Wang, 2002) of spike-based integration, but with the important difference that subthreshold rather than suprathreshold processes are responsible for a rate-limiting integration step.

## STAR★Methods

### Key Resources Table

**Table:** 

REAGENT or RESOURCE	SOURCE	IDENTIFIER
**Antibodies**		

Mouse monoclonal anti-GFP	Memorial Sloan Kettering Monoclonal Antibody Facility	Htz-GFP-19C8
Mouse monoclonal 4F3 anti-discs large	Developmental Studies Hybridoma Bank, University of Iowa	RRID: AB_528203
AlexaFluor633-conjugated goat anti-mouse IgG	Invitrogen (Thermo Fisher)	A-21052; RRID: AB_2535719

**Chemicals, Peptides, and Recombinant Proteins**		

AlexaFluor555-conjugated streptavidin	Invitrogen (Thermo Fisher)	S32355
Biocytin	Sigma-Aldrich	B4261
Paraformaldehyde	Electron Microscopy Sciences	15713
4-methylcyclohexanol	Sigma-Aldrich	153095
Phrixotoxin-2	Alomone labs	P-700
Tetrodotoxin citrate	Tocris	1069
RNaseOUT Recombinant Ribonuclease Inhibitor	Invitrogen (Thermo Fisher)	10777019
cOmplete Protease Inhibitor Cocktail	Roche	11873580001
SuperScript III First-Strand Synthesis SuperMix	Invitrogen (Thermo Fisher)	18080400
LightCycler® 480 SYBR Green I Master	Roche	04707516001
PicoPURE RNA Isolation Kit	Invitrogen (Thermo Fisher)	KIT0204
Protein G Mag Sepharose beads	GE Healthcare	28944008
Vectashield antifade mouting medium	Vector Laboratories	H-1000
TES	Sigma-Aldrich	T5691
NaCl	Sigma-Aldrich	S7653
KCl	Sigma-Aldrich	P9333
NaHCO_3_	Sigma-Aldrich	S6297
NaH_2_PO_4_	Sigma-Aldrich	S8282
CaCl_2_	Sigma-Aldrich	21115
MgCl_2_	Sigma-Aldrich	M1028
Trehalose	Sigma-Aldrich	T9531
Glucose	Sigma-Aldrich	G7528
Sucrose	Sigma-Aldrich	S0389
HEPES	Sigma-Aldrich	H4034
Phosphate buffered saline tablets	Oxoid	BR0014G
MgATP	Sigma-Aldrich	A9187
Na_3_GTP	Sigma-Aldrich	G6129
BaCl dihydrate	Sigma-Aldrich	529591
EGTA	Sigma-Aldrich	E4378

**Experimental Models: Organisms/Strains**		

*Drosophila, w1118; P{RS5}FoxP*^*5-SZ-3955*^	Kyoto Stock Center; DasGupta et al., 2014	126252
*Drosophila, NP7175-GAL4*	Kyoto Stock Center; Tanaka et al., 2008, Murthy et al., 2008	114120
*Drosophila, NP6024-GAL4*	Kyoto Stock Center; Tanaka et al., 2008	105080
*Drosophila, VT030604-GAL4*	Vienna Drosophila Recource Centre; Kvon et al., 2014	200228
*Drosophila, OK107-GAL4*	Bloomington Drosophila Stock Center; Tanaka et al., 2008	854
*Drosophila, UAS-EGFP-L10a*	Gift from R. Jackson; Huang et al., 2013	N/A
*Drosophila, UAS-CD8::GFP*	Bloomington Drosophila Stock Center; Lee and Luo, 1999	32186
*Drosophila, UAS-GCaMP6m*	Bloomington Drosophila Stock Center; Chen et. al., 2013	42748
*Drosophila, UAS-GFP-Shal*	Gift from S. Tsunoda; Diao et al., 2010	N/A
*Drosophila, UAS-DN-Shal*	Gift from S. Tsunoda; Ping et al., 2011	N/A
*Drosophila, UAS-mCherry-CAAX*	Bloomington Drosophila Stock Center; Kakihara et al., 2008	59021
*Drosophila,* Canton-S	Bloomington Drosophila Stock Center	64349
*Drosophila, tubP-GAL80*^*ts*^	Gift from M. Ramaswami and R. Davis; McGuire et al., 2003	N/A

**Oligonucleotides**		

Primer list	Table S5	N/A

**Software and Algorithms**		

Igor Pro	Wavemetrics	https://www.wavemetrics.com
NeuroMatic	NeuroMatic	http://neuromatic.thinkrandom.com
MATLAB	Mathworks	https://www.mathworks.com/
LabVIEW	National Instruments	http://www.ni.com/en-us.html
ImageJ	NIH	https://imagej.nih.gov
pClamp 10	Molecular Devices	https://www.moleculardevices.com/
Prism	GraphPad	https://www.graphpad.com

**Other**		

Borosilicate glass capillaries	Sutter Instruments	BF150-86-10

### Contact for Reagent and Resource Sharing

Requests for resources and reagents should be directed to and will be fulfilled by the Lead Contact, Gero Miesenböck (gero.miesenboeck@cncb.ox.ac.uk).

### Experimental Model and Subject Details

Experimental flies were heterozygous for all transgenes, including *UAS-FoxP*^RNAi^ (Vienna *Drosophila* Recource Centre [VDRC] ID 15732; Dietzl et al., 2007), and homozygous for either a wild-type (*WT*) or the mutant *FoxP*^*5-SZ-3955*^ allele (*w*^*1118*^*; P{RS5}FoxP*^*5-SZ-3955*^, Kyoto Stock Center), as indicated. For translating ribosome affinity purification (TRAP) and patch-clamp recordings, *NP7175-GAL4* (Aso et al., 2009, Murthy et al., 2008, Tanaka et al., 2004; 2008) or *VT030604-GAL4* (Kvon et al., 2014) (VDRC ID 200228) were used to target the expression of *UAS-EGFP-L10a* (Huang et al., 2013) or *UAS-CD8::GFP* (Lee and Luo, 1999) to αβ_c_ or α’β’ KCs, respectively (Figure S1). In functional imaging experiments, where the α’ lobe of the mushroom body could be visually identified, KC populations expressed *UAS-GCaMP6m* (Chen et al., 2013) under the control of *OK107-GAL4* (Tanaka et al., 2008) or *NP7175-GAL4* (Figures S1 and S2). To examine the subcellular distribution of Shal or the behavioral consequences of altered Shal currents, the strong *NP6024-GAL4* line (Tanaka et al., 2008) was used to drive high-level expression of *UAS-GFP-Shal* (Diao et al., 2010) or *UAS-DN-Shal* (Ping et al., 2011) in αβ_c_ KCs. In Figure 4C, αβ_c_ KCs coexpressed *UAS-mCherry-CAAX* (Kakihara et al., 2008); in Figures 6C and 6D, flies bearing *UAS-GFP-Shal* also carried *tubP-GAL80*^*ts*^ (McGuire et al., 2003) to enable the temperature-controlled overexpression of Shal.

Fly strains were cultivated on standard cornmeal agar under a 12 h light:12 h dark cycle at 25°C unless they expressed GAL80^ts^; in this case, the experimental animals and all relevant controls were raised at 18°C.

### Method Details

#### Translating Ribosome Affinity Purification

For each biological replicate, the heads of 400 male and female flies expressing *UAS-EGFP-L10a* (Huang et al., 2013) in the KC population of interest were collected at 7–8 days post-eclosion and homogenized in 500 μl of extraction buffer (pH 7.3) containing 20 mM HEPES, 150 mM KCl, 5 mM MgCl_2_, 0.5 mM dithiothreitol, 1% (v/v) Triton X-100, 100 μg/ml cycloheximide, 100 U/ml RNaseOUT (Invitrogen), and 1 × cOmplete Protease Inhibitor (Roche). Lysates were centrifuged at 14,000 *g* for 15 min at 4°C, and the supernatants were applied to 25 μl Protein G Mag Sepharose beads (GE Healthcare) coated with monoclonal mouse anti-GFP antibody (Htz-GFP-19C8, Memorial Sloan Kettering Monoclonal Antibody Facility). After incubation for 1 h at 4°C, unbound material was collected, and the beads were washed 5 times with 500 μl each of buffer (pH 7.4) containing 20 mM HEPES, 150 mM KCl, 5 mM MgCl_2_, 0.05% (v/v) Triton X-100, and 40 U/ml RNaseOUT.

#### Reverse Transcription and Quantitative Real-time PCR

RNA was isolated from immunoprecipitated ribosomes and unbound material using the PicoPURE RNA Isolation Kit (Life Technologies); 10–15 ng of RNA were then reverse-transcribed using SuperScript III First-Strand Synthesis SuperMix (Invitrogen). cDNA was pre-amplified by multiplex PCR using primers specific for three housekeeping genes (*Gpdh*, *Tbp*, and *Ef1α100E*); *GFP*, *Fas2*, or *trio* as markers for αβ or α’β’ KCs (Awasaki et al., 2000, Grenningloh et al., 1991), respectively; and the *FoxP* isoforms and ion channel genes of interest (Table S5). Transcript levels were determined by quantitative real-time PCR on a LightCycler 480 system (Roche) using SYBR Green I Master Mix (Roche) in 10 μl reactions containing 100 nM of each gene-specific primer and 50 ng of pre-amplified cDNA. All samples were run in technical triplicates; non-reverse-transcribed mRNA and water served as negative controls. Melting curves were analyzed after amplification, and amplicons were visualized by agarose gel electrophoresis to confirm primer specificity. Relative transcript levels were estimated with the help of the 2^-ΔΔ*C*t^ method (Livak and Schmittgen, 2001), using the geometric mean of the *C*_t_ values of the three housekeeping genes for normalization.

#### Functional Imaging

Male flies aged 7 days were fixed to a custom mount with soft thermoplastic wax (Agar Scientific). Cuticle, adipose tissue, and trachea were surgically removed in a window large enough to expose the dorsal brain and antennal nerves, and the preparation was superfused with extracellular solution (pH 7.45) containing 5 mM HEPES, 140 mM NaCl, 2 mM KCl, 4.5 mM MgCl_2_, and 1.5 mM CaCl_2_. Both antennal nerves were cut as far distally as possible, and the antennae were removed to facilitate access. One nerve was aspirated into a fire-polished suction electrode (40 μm bore diameter) to achieve a 1.5 ± 0.3 MΩ seal. A constant voltage stimulator (Digitimer) was used to deliver trains of 1 ms pulses of 2 V at increasing frequencies for 500 ms, with pauses of 20 s between stimulus trains. The pipette resistance was monitored throughout to guarantee constant stimulation currents.

GCaMP6m fluorescence was recorded by two-photon laser scanning microscopy. Excitation light pulses with 140 fs duration and a center wavelength of 910 nm (Chameleon Ultra II, Coherent) were intensity-modulated with the help of a Pockels cell (302RM, Conoptics) and focused by a 20 ×, 1.0 NA water immersion objective (W-Plan-Apochromat, Zeiss) on a Movable Objective Microscope (Sutter Instruments). Emitted photons were separated from excitation light by a series of dichromatic mirrors and dielectric and colored glass filters and detected by a GaAsP photomultiplier tube (H10770PA-40 SEL, Hamamatsu Photonics). Photocurrents were passed through a high-speed amplifier (HCA-4M-500K-C, Laser Components) and a custom-designed integrator circuit to maximize the signal-to-noise ratio (Shang et al., 2007). The microscope was controlled through ScanImage (Vidrio Technologies) via a PCI-6110 DAQ board (National Instruments). Images were acquired at a resolution of 256 × 256 pixels and a frame rate of 5 Hz. All experiments were carried out at room temperature (21–23°C).

*ΔF/F* traces in manually defined regions of interest were calculated in ImageJ by dividing the background-corrected fluorescence by the background-corrected pre-stimulus signal (Lin et al., 2014). To measure the maximal GCaMP6m fluorescence, Ca^2+^ influx was induced at the end of each experiment by rapidly applying KCl to the bath at a final concentration of 100 mM (Figure S2).

#### Structural Imaging

Brains of male flies were dissected 2 days after eclosion and mounted in phosphate-buffered saline (PBS; 137 mM NaCl, 3 mM KCl, 8 mM Na_2_HPO_4_, 1.5 mM KH_2_PO_4_, pH 7.3) for imaging native fluorescence (Figure 4C). For immunostaining (Figures 2A, 2J, and S1), dissected brains were fixed in 4% (w/v) paraformaldehyde in PBS for 20 min at room temperature, washed four times for 20 min in PBS, and permeabilized and blocked in PBS containing 0.2% (v/v) Triton X-100 and 5% (v/v) goat serum for 1 h. To label synaptic structures, the samples were first incubated with mouse monoclonal anti-discs large antibody 4F3 (Developmental Studies Hybridoma Bank, University of Iowa, 1:50) and then with Alexa Fluor 633-conjugated goat anti-mouse IgG (A-21052, Invitrogen, 1:200). Each incubation lasted for 48 h at 4°C and was followed by four 20 min washes in PBS. Stained samples were mounted in Vectashield (Vector Labs) and imaged on a Leica TCS SP5 confocal microscope equipped with an HCX PL APO 40 ×, 1.3 CS oil immersion objective (Leica). Images were processed in ImageJ.

#### Electrophysiology

For whole-cell patch-clamp recordings *in vivo*, male flies aged 6–24 h post-eclosion were prepared as for functional imaging, but the perineural sheath was also removed to provide access to KC somata while the antennae and antennal nerves were left intact for eliciting odor responses. The brain was continuously superfused with extracellular solution (pH 7.3) containing 5 mM TES, 103 mM NaCl, 3 mM KCl, 26 mM NaHCO_3_, 1 mM NaH_2_PO_4_, 1.5 mM CaCl_2_, 4 mM MgCl_2_, 8 mM trehalose, 10 mM glucose, and 7 mM sucrose (275 mOsM, equilibrated with 5% CO_2_ and 95% O_2_). Patch pipettes (15–17 MΩ) were fabricated from borosilicate glass capillaries with outer and inner diameters of 1.5 and 0.86 mm (Sutter Instruments), using a PC-10 micropipette puller (Narishige), and filled with solution (pH 7.3) containing 10 mM HEPES, 140 mM potassium aspartate, 1 mM KCl, 4 mM MgATP, 0.5 mM Na_3_GTP, 1 mM EGTA, and 10 mM biocytin (265 mOsM). Pipettes were visually targeted to green-fluorescent KC somata using a combination of epifluorescence and differential interference contrast on an Axioskop 2 FS mot microscope (Zeiss) equipped with a 60 ×, 1.0 NA water-immersion objective (LUMPLFLN60XW, Olympus) and an X-Cite 120PC Q light source (Excelitas Technologies). αβ_c_ KCs labeled by *NP7175-GAL4* were considered a homogeneous population and sampled randomly, regardless of their discernible origin from one of the four neuroblast clones that give rise to the mushroom bodies (Ito et al., 1997, Tanaka et al., 2008) (Figure 2A). Estimates of the number of αβ_c_ KCs captured by *NP7175-GAL4* range from 58 (Tanaka et al., 2004) to 92 (Murthy et al., 2008) to 203 cells per hemisphere (Aso et al., 2009) and thus agree with our estimate of ∼80 FoxP-positive αβ_c_ KCs per hemisphere to differing degrees (DasGupta et al., 2014). Should the high estimate prove accurate, the population of αβ_c_ KCs we characterized may include FoxP-negative cells.

Signals were acquired at room temperature (21–23°C) with a MultiClamp 700B amplifier (Molecular Devices), lowpass-filtered at 10 kHz, and sampled at 50 kHz using a Digidata 1440A digitizer controlled through pCLAMP 10 (Molecular Devices). Data were corrected for liquid junction potential (Neher, 1992) and analyzed with custom procedures, using the NeuroMatic package (http://neuromatic.thinkrandom.com) in Igor Pro (WaveMetrics).

In current-clamp recordings, bridge balance was used to compensate for series resistance. The most negative membrane potential recorded immediately after break-in, in the absence of a holding current and without correcting for errors introduced through seal conductances, was taken to represent the resting potential. Because of the high input resistances of KCs (Turner et al., 2008) (Figures 2G and 2P), the true resting potentials are likely to be more hyperpolarized (Gouwens and Wilson, 2009). Only cells with a measured resting potential below –35 mV and a spiking response to depolarizing current injections were characterized further. Spikes were detected by finding minima in the time derivative of the membrane potential trace, which allowed the distinction of action potentials from EPSPs on the basis of differences in the slopes of membrane repolarization. Spike thresholds and spike latencies were estimated by recording voltage responses to 600 ms current ramps, from –2 to +20 pA, starting at a membrane potential of –75 ± 2 mV. Input resistances were calculated from linear fits of the steady-state voltage changes elicited by 750 ms steps of hyperpolarizing currents (1 pA increments, starting at –4 pA) from a pre-pulse potential of –70 ± 5 mV. Membrane time constants were determined by fitting a single exponential to the voltage deflection caused by a hyperpolarizing 2.5 pA current step lasting 750 ms. Action potential waveforms represent averages of individual, baseline-subtracted spikes recorded at membrane potentials just above threshold, when neurons fired at ≤ 5 Hz; EPSP waveforms represent averages of individual, baseline-subtracted EPSPs occurring spontaneously at a membrane potential of –70 ± 5 mV. Decay time constants were determined by fitting single exponentials to the averaged EPSP waveforms.

For electrical stimulation of the antennal nerve, both antennal nerves were cut as far distally as possible. One nerve was aspirated into a fire-polished suction electrode (40 μm bore diameter) to achieve a 1.5 ± 0.3 MΩ seal. The amplitude of 50 μs voltage pulses delivered by a constant voltage stimulator (Digitimer) was gradually increased until EPSCs or EPSPs could be detected in the recorded KC; the stimulus intensity was then further increased by ∼25% to minimize the fraction of transmission failures. Once a stable response was obtained, the stimulus intensity was left unchanged for the remainder of the experiment. The coefficient of variation (CV) of EPSC amplitudes within a trial was 0.440 ± 0.019 (mean ± SEM, n = 22 trials), comparable to that attributed to quantal variability at individual excitatory synapses (McAllister and Stevens, 2000). Consistent with this interpretation, the variation of mean evoked EPSC amplitudes between trials was smaller than the variation within trials (CV = 0.134 ± 0.014; mean ± SEM, n = 3 cells).

Following a first set of stimulation trials at a range of frequencies, BaCl_2_ was added to the extracellular solution at a final concentration of 150 μM, and a second set of stimulation trials was conducted; the ability of 150 μM Ba^2+^ to block Shal currents was verified in voltage-clamp recordings (Figures 3E and 3F). In some experiments, a third and final set of antennal nerve stimulation trials was performed after the addition of 1 μM tetrodotoxin (TTX, Tocris) to block voltage-gated Na^+^ channels and ensure that the responses of the recorded KC were synaptically mediated.

For olfactory stimulation (Shang et al., 2007), mass flow-controlled streams of MCH-infused air (CMOSens, Sensirion) were directed at the fly’s head through a behavioral chamber cut in half; flow rates matched those in behavioral experiments (0.25 l/min). Solenoid valves (The Lee Company) under LabVIEW control (National Instruments) switched between odor streams containing basal (2–18 ppm) and peak (20 ppm) MCH concentrations. Steady-state odor concentrations were calibrated using a ppbRAE 3000 photoionization detector (RAE systems); the kinetics of MCH concentration changes at the position of the fly were recorded with a 200B miniPID (Aurora Scientific) at 10 kHz and smoothed with a Gaussian filter. Pressure changes caused by the opening and closing of valves were balanced with the help of a set of pressure-compensating valves and monitored periodically with a mass flow sensor (FBAL001DU, Sensor Technics). The membrane potential at the beginning of each MCH stimulation trial was –50 ± 5 mV. KCs were classified according to their spiking responses to a high-contrast stimulus (MCH concentration ratio 0.1) (Figure 7B). Up cells fired spikes during the rising phase of the stimulus; down cells responded during the falling phase; unresponsive cells did not show a spiking response to MCH concentration changes in either direction. Membrane potential variances as functions of base MCH concentration were quantified in the 5 s window immediately before stimulus onset.

Voltage-clamp recordings, except those in Figures 3E, 3F, 5B, and S3A–S3D, were obtained in the presence of 1 μM TTX; because KCs lack significant Ca^2+^ currents (Gasque et al., 2005), no measures were taken to block Ca^2+^ channels. Series resistances were monitored but not compensated and allowed to rise by at most 20% during the course of a recording. Capacitative transients and linear leak currents were subtracted using a P/4 protocol. Steady-state activation parameters were determined by applying depolarizing 50 ms voltage pulses from a holding potential of –100 mV; the pulses covered the range to +50 mV in steps of 10 mV. A-type current densities were determined by dividing the peak outward current during the first 10 ms of the test pulse by the cell’s capacitance after digitally subtracting non-inactivating outward currents. Membrane capacitances were estimated by integrating the capacitative currents produced by 10 ms steps of 20 mV and found to be identical among genotypes (p = 0.3127, Kruskal-Wallis test), averaging 2.41 ± 0.07 pF (n = 81 cells), 2.45 ± 0.07 pF (n = 49 cells), and 2.49 ± 0.06 pF (n = 42 cells) for αβ_c_ KCs of wild-type, *FoxP*^*5-SZ-3955*^, and *FoxP*^RNAi^ flies, respectively (means ± SEM). Steady-state inactivation parameters were obtained with the help of a two-pulse protocol, in which a 500 ms pre-pulse (–120 to –30 mV in 5 mV increments) was followed by a 50 ms test pulse to +50 mV; non-inactivating outward currents, measured from a pre-pulse potential of –30 mV, were subtracted. Peak A-type currents (*I*_A_) were normalized to the maximum current amplitude (*I*_max_) of the respective cell and plotted against the pre-pulse potential (*V*). Curves were fit to the Boltzmann function $I_{A}/I_{\max}=1/\left(1+e^{\left(V-V_{0.5}\right)/k}\right)$ to determine the half-maximal inactivation voltage (*V*_0.5_) and slope factor (*k*). To estimate the inactivation time constant, single exponential functions were fit to the decaying phase of currents elicited by 100 ms depolarizing voltage pulses after digitally subtracting non-inactivating outward currents. Phrixotoxin-2 (Alomone Labs) was dissolved freshly in extracellular solution at 100 μM and applied directly to the soma of the recorded cell, using a patch pipette (3–4 MΩ) connected to a pneumatic drug ejection system (5 psi, PEDS-02DX, npi electronic).

#### Measurement of Reaction Times and Decision Accuracies

Male flies aged 7–8 days were analyzed individually in transparent plexiglass chambers (50 mm long, 5 mm wide, 1.3 mm high) (Claridge-Chang et al., 2009, DasGupta et al., 2014). Two independently controlled odor streams entered the chambers through ports at the ends of the long chamber axis, converged at the center, and left through lateral vents (Figure 6A). Printed circuit boards, connected via solid-state relays (Fairchild HSR312L) to a 70 V power supply, served as floors and ceilings. Filtered, flow-controlled (CMOSens, Sensirion), and humidified carrier air was mixed, in the indicated ratios, with flow-controlled odor streams drawn through vials containing MCH, yielding a final flow rate of 0.25 l/min per half-chamber. Twenty chambers were operated simultaneously in an incubator (Sanyo MIR-154) at 25°C. The chambers were backlit by 940 nm LEDs (TSAL6100, Vishay) and imaged using a Stingray F080B CCD camera (Allied Vision Technologies) with an 18 mm lens (Edmund Scientific). A virtual instrument written in LabVIEW (National Instruments) controlled the delivery of odors and electric shock and recorded the positions of the 20 flies as functions of time (Claridge-Chang et al., 2009).

Flies were trained to avoid 20 ppm MCH by pairing twelve consecutive odor presentations with electric shock (DasGupta et al., 2014). During a 2 min testing period immediately after training, one half of each chamber was filled with MCH at the reinforced concentration while the other half was perfused with a lower concentration; the concentration ratio was titrated to determine the difficulty of the perceptual task (DasGupta et al., 2014).

Experimental data were processed offline in MATLAB (The MathWorks) (DasGupta et al., 2014). Position measurements were smoothed by computing 0.5 s moving averages and filtered to exclude animals making < 2 end-to-center runs per testing period. Each chamber contained a central, 7 mm wide decision zone (Figure 6A). Correct and incorrect choices during the test period were combined into individual accuracy scores, which were used to calculate population averages. The time between entry into and exit from the decision zone was recorded as the reaction time (DasGupta et al., 2014). A fly’s trajectory within the decision zone, viewed along the long chamber axis (Figures 6B–6D), typically consisted of an entry phase at high velocity, a pause at the odor interface, and an accelerating exit phase. Reaction times were normalized to each fly’s instantaneous walking speed, estimated as the average speed from the point of entry into the decision zone to the first reduction to 10% of the entry velocity (DasGupta et al., 2014). In Figures 6C and 6D, all flies were kept at 31°C for 40–48 h before the experiment to allow the temperature-induced overexpression of Shal.

#### Drift-diffusion Model

Accuracy and reaction time measurements (DasGupta et al., 2014) were fit to a drift-diffusion model (Bogacz et al., 2006). The model assumes that the difference in evidence favoring one alternative over the other evolves as a result of drift *vdt* and Gaussian diffusion noise with zero mean and variance *σ*^*2*^*dt* until one of two symmetrical decision criteria (a.k.a. bounds) located at ± *A* is reached. Genotype-specific scaling factors *k*_*i*_ relate the drift rates *v*_*i,x*_ linearly to the difficulty *x* of odor intensity discrimination, which we quantified as (1–odor concentration ratio): *v*_*i,x*_ = *k*_*i*_ *x*. Solving the first-passage problem for the decision variable yields analytical expressions (Bogacz et al., 2006) that link the observed accuracies (fractions of correct choices *F*_*i,x*_, normalized by the empirically determined maximal accuracy *F*_max_ = 0.9905) and reaction times *T*_*i,x*_ to genotype (via the scaling factors *k*_*i*_ and noise variances *σ*_*i*_^*2*^), task difficulty *x*, criterion height *A*, and residual time *T*_0_:

(1) $$F_{i,x}=F_{\max}\frac{1}{1+e^{-2A\frac{k_{i}x}{\sigma_{i}^{2}}}}\text{,}$$

(2) $$T_{i,x}=\frac{A}{k_{i}x}\tanh\left(A\frac{k_{i}x}{\sigma_{i}^{2}}\right)+T_{0}\text{.}$$

In order for accuracy to become independent of genotype (that is, independent of the genotype-specific parameters *k*_*i*_ and *σ*_*i*_^*2*^), as is observed in Figure 7I (DasGupta et al., 2014), the noise variances *σ*_*i*_^*2*^ must be proportional to the corresponding scaling factors: *k*_*i*_ = *c σ*_*i*_^*2*^, where *c* is a constant. Equations (1) and (2) then simplify to:

(3) $$F_{x}=F_{\max}\frac{1}{1+e^{-2A^{'}x}}\text{,}$$

(4) $$T_{i,x}=\frac{A^{'}}{k_{i}^{'}x}\tanh\left(A^{'}x\right)+T_{0}\text{.}$$

The new parameters *A’* and *k’*_*i*_ absorb the proportionality constant *c*: *A’ = c A* and *k’*_*i*_ *= c k*_*i*_. The free parameters *A’*, *k’*_*WT*_, *k’*_*FoxP*_, and *T*_*0*_ were estimated from a simultaneous least-squares fit, computed with the help of the Nelder-Mead simplex algorithm implemented in MATLAB, of accuracy and reaction time data of wild-type and *FoxP*^*5-SZ-3955*^ mutant flies (DasGupta et al., 2014) to Equations (3) and (4). The model returned a residual time *T*_*0*_ of 1.296 s, a bound height *A’* of 4.383, and genotype-specific scaling factors *k’*_*i*_ of 12.659 for wild-type and 6.704 for *FoxP*^*5-SZ-3955*^ mutant flies. The units of *A’* and *k’*_*i*_ are arbitrary.

### Quantification and Statistical Analyses

Data were analyzed in Prism 6 (GraphPad). Group means were compared by one- or two-way ANOVA, using repeated-measures designs where appropriate, followed by post hoc analyses using Holm–Šídák’s multiple comparisons test. Where the assumptions of normality or equality of variances were violated (as indicated by Shapiro–Wilk and Brown–Forsythe tests, respectively), group means were compared using two-sided Kruskal-Wallis test, followed by Dunn’s multiple comparisons test. Reaction time distributions were compared by Kolmogorov–Smirnov test, using the Bonferroni correction to adjust the level of statistical significance; stimulus-response and current-spike frequency curves were analyzed by *F* test.
