# Supplementary material for: Dendritic Integration of Sensory Evidence in Perceptual Decision-Making
Source: Cell. 2018 May 3;173(4):894–905.e13. doi: 10.1016/j.cell.2018.03.075 (PMC5947940; doi:10.1016/j.cell.2018.03.075)
Supplement: Document S1. Tables S1–S5 [file mmc1.pdf]

Cell, Volume *173*

Supplemental Information

Dendritic Integration of Sensory Evidence in Perceptual Decision-Making

Lukas N. Groschner, Laura Chan Wah Hak, Rafal Bogacz, Shamik DasGupta, and Gero Miesenböck

Table S1. Numbers of Biological Replicates of qPCR Measurements, Related to Figures 1, 4, and S5

	Genotype					
	NP7175-GAL4/+; UAS-EGFP-L10a/+ ($\alpha\beta_c$)			*UAS-EGFP-L10a/VT030604-GAL4* ($\alpha'\beta'$)		
Transcript	*WT*	*FoxP*$^{5\text{-}SZ\text{-}3955}$	*FoxP*^{RNAi}	*WT*	*FoxP*$^{5\text{-}SZ\text{-}3955}$	*FoxP*^{RNAi}
FoxP-RC	16	15	16	4	4	4
FoxP-RD	16	16	16	4	4	4
Shaker	10	9	7	9	7	7
Shal	12	10	7	7	6	7
Shaw	10	12	10	8	7	7
Shab	9	9	8	7	7	6
Shawl	8	8	10	6	6	6
KCNQ	8	7	7	6	6	6
eag	9	8	8	7	7	8
elk	8	7	8	6	5	6
sei	8	7	8	6	6	5
SK	8	10	6	8	8	7
Slo	8	7	8	7	6	7
Irk1	9	9	8	7	7	7
Irk2	10	10	8	6	5	8
Task6	10	10	10	6	7	6
Task7	9	8	9	7	6	8
Ork1	10	9	9	8	9	8
sand	8	11	10	8	8	9
CG42594	10	11	11	8	8	7
CG9194	12	10	10	8	8	7
CG42346	9	8	8	8	8	7
CG1688	10	9	9	8	8	8
CG10864	7	7	8	8	8	7
CG34396	10	9	8	7	7	7
Caα1D	8	8	8	6	7	6
Caα1T	8	8	8	6	6	6
cac	8	8	8	6	9	6
para	8	7	8	7	6	6
na	8	8	8	7	6	6
Clc	8	7	7	7	7	7
HisCl1	8	8	8	7	7	7
Rdl	8	7	8	6	5	6
trp	8	7	8	6	6	7

Table S2. Sample Sizes for Behavioral Measurements, Related to Figure 6

Genotype	Concentration ratio							
	0.1		0.4		0.7		0.9	
	Flies	Decisions	Flies	Decisions	Flies	Decisions	Flies	Decisions
NP6024-GAL4/+; +; FoxP$^{5\text{-}SZ\text{-}3955}$	168	550	110	363	156	635	179	665
UAS-DN-Shal/+; FoxP$^{5\text{-}SZ\text{-}3955}$	152	539	127	446	151	665	203	913
NP6024-GAL4/+; UAS-DN-Shal/+; FoxP$^{5\text{-}SZ\text{-}3955}$	112	371	118	398	165	727	144	619
NP6024-GAL4/+	188	530	119	420	144	666	110	480
UAS-GFP-Shal/+; tubP-GAL80^{ts}/+	141	410	171	643	134	706	110	547
NP6024-GAL4/+; UAS-GFP-Shal/+; tubP-GAL80^{ts}/+	118	306	148	410	101	401	133	575
VT030604-GAL4/+	83	182	114	303	179	652	177	617
UAS-GFP-Shal/+; tubP-GAL80^{ts}/+	80	187	139	446	151	665	121	486
UAS-GFP-Shal/+; VT030604-GAL4/tubP-GAL80^{ts}	82	160	130	433	143	601	119	385

Table S3. Sample Sizes for Neurometric Measurements, Related to Figure 7

Genotype	Concentration ratio							
	0.1		0.4		0.7		0.9	
	KCs	Trials	KCs	Trials	KCs	Trials	KCs	Trials
NP7175-GAL4/+; UAS-CD8::GFP/+ (WT)	13	66	11	73	9	44	7	36
NP7175-GAL4/+; UAS-CD8::GFP/+; FoxP$^{5-SZ-3955}$	8	52	7	39	8	41	7	48
UAS-CD8::GFP/+; VT030604-GAL4/+	4	33	4	23	4	22	4	24

Table S4. Sample Sizes for Neurometric Measurements, Related to Figure S7

Stimulus protocol	Concentration ratio							
	0.1		0.4		0.7		0.9	
	KCs	Trials	KCs	Trials	KCs	Trials	KCs	Trials
One 10-s intensity step	5	27	5	21	4	21	4	23
Ten intensity cycles (1 Hz)	5	25	5	17	4	19	4	18

Table S5. Primer Sequences, Related to Figures 1, 4, and S5

Gene	Forward primer (5' - 3')	Reverse primer (5' - 3')
Gpdh	CTTCTTCAGCGACACCCATT	ACCGAACTCGTTGTCGTACC
Tbp	ACAGGGGCAAAGAGTGAGG	CTTAAAGTCGAGGAACTTTGCAG
Ef1α100E	GCGTGGGTTTGTGATCAGTT	GATCTTCTCCTTGCCCATCC
GFP	CACATGGTCCTTCTTGAGTTTG	CGACGCCATTTTGTATAGTTCA
Fas2	CGCCATCGGCTATTCATC	CTTGGATGACGTCAATTCCTC
trio	CAGCTACCTGATCAGCATTCC	GCATGTCAATGATGACTGTGAA
FoxP-RC	GCCACTTGACAATCGATGAA	TCCGAATCCGAACCTTCTTA
FoxP-RD	CAATTCGCCGGACACTAACT	CAAAACGGAAGGAGTTTGGA
Shaker	TTAAGTGTTTCCCGACTTGCC	CTTTTTCTCTCTCTGCACGTGC
Shal	GATGAGGTGGGAAAAGTTGC	TGAACTTATTGCTGTCATTTTGC
Shaw	AAGCGGAGACGAGTGCTTC	GGTACCGCAACCACTGACA
Shab	CGACAATTGACGCGATGTAG	TTGAAGCGGTGTTGATTCAC
Shawl	ACGACTGGCCACGACTTC	TGCTGATCCTTGCGTTCC
KCNQ	CCTCTGGTGGGGTGTGAT	GCGAAAAAGGATATTCCCAGA
eag	CAGCAGGATCAGTTCGAGATT	TTGCCAAACTGCGAGAGAG
elk	TAGCGCCCCAGAACACAT	CCGAGCACAAAGTTCGAGT
sei	AGTTCATTCGCTTTCACCAGA	ATAGGTCCAGGCGTGCTG
SK	ACTCAGACTACCCTAGGTATGAAATGT	TGTGTTTGGCCGAGTTTGT
Slo	CACCTTTATGACAGAAGCGAAA	ATGAGGGATGCAATGCTGA
Irk1	AGGAACGCTTCGAAGTGGT	ATGACGCCCTCTGAAATCGAT
Irk2	CTTGCCAGAAAACCAAGAGG	CCAATAGTGTGCTGCGTTTC
Task6	TTACGATGAATACCGAGGATGA	GTTATCACATCGCCCTCCAG
Task7	TTGTATGGGCTATGCTATGGTG	TCAGACGTTCTCCGATAGACTG
Ork1	GGCAATCTATTACCATATTGAGCA	TGTGTGGTCGTATTCTTGTCG
sand	CGTCACGGGTGAGCATCT	TCCGGCTACCTATCGCTGT
CG42594	GCTGCTTCTCGCCTACACA	CTTGGTGCTGGCCAGAGT
CG9194	CGAATCAGAAGGCGATCAG	GACATGGTCACCCTCCTCTG
CG42346	CTGCTACAAGTTCGAGGATGG	CCGATATTCTCTCGAGTGTTGA
CG1688	GCTAATTGCTCAATGGCGTTA	ATTGTTCAGCAGCGCAGAG
CG10864	ATGTGGGCAGAAGTCCAGAG	CGACAGGCAGAACATTAGGG
CG34396	TGTATTCCGCACAATCGAAG	TGAAGAAACTCTTTTGCTGATCC
Caα1D	GGCGAATGCCATTAACTATGA	TCGATTGGATCATGCGTTC
Caα1T	CTGATGCTCATCACCAATCC	GCTGATACATGCCGAGAGTG
cac	GGTTTGGCCAACTCGATTC	TCCTGCGACGGATGTCTC
para	AACAACATCAGCGGATAGAGC	CGCCATGTTCGATTAGC
na	CGTGGTGGTCCTGATCAATAG	CGTATGCACCTCTCCCTTG
Clc	AAAATACTCGAAAAGCGACGA	TCCATGATGATACCCGCTTC
HisCl1	CATCAGTGATCTGGACGACCT	TATAAATGGCTGTGGCATGG
Rdl	GCATCCGGGAAAAGTAAAAA	TGCGTGAGTACTTGTCGATGT
trp	CGAAAAGAAGCCACTGGATG	TGCAGCCAACATAGAAGCTC
